# Why Hand–Wrist Bandaging Could Improve Performance in Elite Soccer Players? A Scoping Review on the Biomechanical Rationale of Upper Limb Role in Kicking

**DOI:** 10.3390/sports14050189

**Published:** 2026-05-06

**Authors:** Rocco De Vitis, Luca Lombardi, Matteo Guzzini, Arturo Militerno, Giuseppe Taccardo, Marco Passiatore

**Affiliations:** 1 Department of Orthopedic and Geriatric Sciences, Fondazione Policlinico Universitario Agostino Gemelli IRCCS (Istituto di Ricovero e Cura a Carattere Scientifico), 00168 Roma, Italy; arturo.militerno@policlinicogemelli.it (A.M.); giuseppe.taccardo@guest.policlinicogemelli.it (G.T.); 2 Independent Researcher, Fisioterm Rehabilitation Center, 00189 Rome, Italy; lucalombardi@fisioterm.com; 3 Department of Orthopaedics, UniCamillus International Medical University, 00131 Rome, Italy; matteo.guzzini@unicamillus.org; 4 Department of Bone and Joint Surgery, University of Brescia, 25123 Brescia, Italy; marco.passiatore@unibs.it

**Keywords:** soccer kicking, biomechanics, upper limb, hand–wrist bandage, proprioception, proximal-to-distal sequencing, athletic performance

## Abstract

Background: Soccer kicking biomechanics has traditionally focused on lower limbs, overlooking whole-body integration. Three-dimensional motion analyses have demonstrated that upper limbs contribute substantially through tension arc formation, counterbalancing, and kinetic chain coordination. The hand–wrist complex may influence performance through proprioceptive pathways, yet this remains untested. Methods: Following PRISMA-ScR guidelines, we searched PubMed/MEDLINE, Web of Science, and SPORTDiscus (inception—February 2026). Peer-reviewed studies examining kicking mechanics, kinetic chains, and joint proprioception were included. Two reviewers independently screened records and extracted data. Narrative synthesis was used to organize findings across four thematic categories: upper limb biomechanics, kinetic chain principles, wrist–hand stability, and proprioceptive enhancement. Results: From 3847 records, 51 studies (1988–2025) were included. Upper limbs are essential for kicking through tension arc formation, energy transfer, and balance maintenance. Kinetic chains operate bidirectionally; available evidence suggests that proximal segment deficits are associated with substantially increased compensatory demands at distal segments. External joint support has been shown to enhance proprioception and force perception. Conclusions: This scoping review identifies a theoretical rationale and a critical research gap: no direct empirical evidence exists that hand–wrist bandaging affects kicking performance. Evidence from adjacent domains (upper limb kicking biomechanics, kinetic chain theory and proprioceptive enhancement with external supports) provides indirect, translational support for the plausibility of a hypothesis that remains entirely untested. Future research should employ within-subject crossover designs in elite soccer players to determine whether this intervention produces any measurable effect. Practical recommendations to athletes or practitioners are premature and are not supported by the current evidence base.

## 1. Introduction

Soccer kicking has been extensively investigated as a predominantly lower limb motor task since the pioneering work of Isokawa and Lees in 1988 [1]. Early research focused on hip flexion velocity, knee extension mechanics, and ankle plantarflexion timing using two-dimensional video analysis [2,3]. This reductionist approach, while generating valuable insights into leg mechanics, did not fully account for the integrated, whole-body nature of human movement. The transition from two-dimensional leg kinematics to three-dimensional full-body biomechanical analysis has substantially advanced our understanding of kicking mechanics [4]. Contemporary investigations employing comprehensive motion capture systems reveal that soccer kicking involves complex coordination across all body segments, with significant and previously underappreciated contributions from trunk rotation, arm movement, and shoulder positioning [5,6].

Modern sports biomechanics emphasizes the kinetic chain concept, which represents the principle that force generation and transfer occur through sequential activation of body segments from proximal to distal [7,8]. Originally articulated by Putnam [9] and extensively developed by Kibler and colleagues [10,11], kinetic chain theory posits that each segment contributes to and influences the performance of adjacent segments through coordinated timing and force transmission. Critically, Kibler’s kinetic chain framework [10], which explicitly includes the hands as terminal segments of force transfer in integrated athletic activities, provides theoretical justification for investigating distal upper limb influences on whole-body athletic performance. This definition provides theoretical justification for investigating distal upper limb influences on whole-body athletic performance.

Recent biomechanical studies demonstrate that upper limbs serve multiple essential functions during soccer kicking. Shan and Zhang [4] identified systematic upper body coordination patterns, termed the tension arc, that form through trunk twisting toward the non-kicking side with contralateral shoulder extension and abduction. Additionally, the tension arc was found to correlate significantly with ball velocity. Biomechanical analyses confirmed that trunk and upper body segments contribute substantially to total kinetic energy in kicking and striking movements [7,12], challenging the conventional view of kicking as exclusively lower limb-driven.

Upper limbs participate in balance and counterbalancing because arms move in opposition to the kicking leg to stabilize the trunk and maintain center of mass control during the critical single-leg support phase [5,13]. Arm movement amplitude correlates with kick velocity, and elite players demonstrate superior coordination between the upper and lower body segments compared to novices [14,15].

If upper limbs contribute meaningfully to kicking performance, then optimizing stability at the distal terminus of the upper extremity may theoretically enhance whole-body coordination and kicking performance through proprioceptive and neuromechanical mechanisms. It is important to note, however, that this remains a speculative hypothesis: the biomechanical evidence reviewed here concerns upper limb contributions to kicking, not the specific effect of hand–wrist stabilization. The inferential leap from one to the other involves substantial assumptions that can only be resolved through direct empirical testing. This hypothesis represents an unexplored frontier in soccer biomechanics. While external joint support via taping and bracing has been extensively studied for lower extremity joints [16,17] and has been demonstrated to enhance proprioception and motor control [18,19], no research has examined whether hand–wrist stabilization influences kicking performance.

It is acknowledged that this reasoning involves extrapolation from adjacent domains, and the specific direction and magnitude of any such effect remain entirely untested. The following review, therefore, adopts a deliberately exploratory approach, mapping indirect evidence rather than asserting established facts.

Given the complete absence of direct research on hand–wrist stabilization effects on soccer kicking, a traditional systematic review with meta-analysis is not feasible. Scoping review methodology, as defined by Arksey and O’Malley [20] and refined according to PRISMA-ScR guidelines [21], is therefore the appropriate design to map existing indirect evidence and systematically identify critical research gaps, without implying the availability of direct experimental evidence.

The aim of this review is to systematically map and synthesize evidence on the role of upper limbs in soccer kicking biomechanics, kinetic chain principles linking proximal stability to distal performance, and mechanisms by which hand–wrist stabilization could theoretically enhance kicking performance. This scoping review addresses three key questions. First, what is the biomechanical role of the upper limbs during soccer kicking, and how do they contribute to performance outcomes? Second, what kinetic chain principles explain relationships between proximal stability and distal performance, and do these relationships operate bidirectionally? Third, what neuromechanical and proprioceptive mechanisms could theoretically link hand–wrist stabilization to enhanced whole-body coordination and kicking performance?

## 2. Materials and Methods

### 2.1. Protocol and Registration

This scoping review follows the methodological framework proposed by Arksey and O’Malley [20] and refined by Levac et al. [22], aligned with the Preferred Reporting Items for Systematic Reviews and Meta-Analyses Extension for Scoping Reviews (PRISMA-ScR) guidelines [21]. The review protocol was not pre-registered. While prospective registration is increasingly encouraged to minimize reporting bias, it is not yet standard or consistently required for scoping reviews, which are by nature exploratory and hypothesis-generating rather than hypothesis-testing [20,21]. This represents a methodological limitation that should be considered when interpreting the findings.

### 2.2. Information Sources and Search Strategy

A comprehensive literature search was conducted across multiple electronic databases from their inception through 21 February 2026. The primary databases included PubMed/MEDLINE (1946 onwards), Web of Science Core Collection (1900 onwards), and SPORTDiscus (1985 onwards). Additionally, Google Scholar was used as a supplementary tool to identify gray literature and potentially relevant sources not indexed in the primary databases. Records identified exclusively through Google Scholar were subject to the same eligibility criteria as records from the primary databases; however, given the non-systematic nature of Google Scholar searching, the precise number of records identified through this source alone cannot be reported with the same precision as the primary database searches. For transparency, the PRISMA-ScR flow diagram reports records from primary databases separately (Supplementary Materials). Google Scholar contributed primarily to the identification of gray literature rather than to the primary peer-reviewed evidence base. We employed a combination of controlled vocabulary terms, specifically Medical Subject Headings (MeSH), where applicable, alongside free-text keywords to maximize retrieval sensitivity. While the fundamental conceptual framework remained consistent across all databases, individual search strategies were adapted to accommodate the specific indexing systems and controlled vocabularies of each database platform.

Our search strategy was structured around five interconnected conceptual domains.

Domain 1: Soccer Kicking Mechanics

(“soccer” OR “football” OR “association football”) AND (“kick*” OR “instep kick” OR “shooting”) AND (“biomechanics” OR “biomechanical” OR “kinematics” OR “kinematic” OR “kinetics” OR “kinetic” OR “motion analysis” OR “movement analysis” OR “gait analysis”)

Domain 2: Upper Limb Movements

(“upper limb” OR “upper extremity” OR “arm” OR “arms” OR “arm swing” OR “arm movement”) AND (“trunk rotation” OR “trunk movement” OR “torso rotation” OR “shoulder” OR “shoulder movement” OR “shoulder mechanics”) AND (“kick*” OR “kicking” OR “coordination”)

Domain 3: Kinetic Chain Principles

(“kinetic chain” OR “kinematic chain”) AND (“proximal-to-distal” OR “proximal to distal” OR “sequential activation” OR “sequencing” OR “segmental coordination” OR “intersegmental coordination” OR “force transfer” OR “energy transfer” OR “summation of speed” OR “multi-joint” OR “multijoint”)

Domain 4: Wrist and Hand Stability

(“wrist” OR “hand” OR “distal segment”) AND (“stability” OR “stabilization” OR “grip strength” OR “hand grip”) AND (“proprioception” OR “proprioceptive” OR “motor control” OR “neuromuscular control” OR “performance” OR “kick* performance”)

Domain 5: External Support Interventions

(“taping” OR “tape” OR “athletic tape” OR “kinesio tape” OR “bracing” OR “brace” OR “orthosis” OR “orthotic” OR “bandage” OR “bandaging” OR “external support”) AND (“proprioception” OR “proprioceptive feedback” OR “neuromuscular” OR “sensorimotor” OR “motor control” OR “stability”)

Combined Comprehensive Search String

((“soccer” OR “football” OR “association football”) AND (“kick*” OR “instep kick” OR “shooting”)) AND ((“upper limb” OR “arm” OR “trunk rotation” OR “shoulder”) OR (“kinetic chain” OR “proximal-to-distal” OR “segmental coordination”) OR (“wrist” OR “hand” OR “grip strength”) OR (“taping” OR “bracing” OR “orthotic”))

The first domain focused on soccer kicking mechanics, incorporating terms related to soccer or association football combined with kicking actions and biomechanical analysis, including both kinematic and kinetic parameters. The second domain addressed the role of upper limb movements, encompassing terminology related to arm movement, trunk rotation, and shoulder mechanics in relation to kicking coordination. The third conceptual area targeted kinetic chain principles, specifically the proximal-to-distal sequencing pattern, segmental coordination, force transfer mechanisms, and the summation of speed concept that characterizes efficient multi-joint movements.

The fourth domain explored wrist and hand stability mechanisms, integrating terms related to distal segment stabilization, grip strength, and their relationship to proprioceptive feedback, motor control, and overall performance outcomes. Finally, the fifth domain examined external support interventions, including various forms of athletic taping, bracing, bandaging, and orthotic devices, with particular attention to their effects on proprioceptive input, neuromuscular function, and sensorimotor control. These conceptual domains were systematically combined using appropriate Boolean operators to create a comprehensive search framework that captured the multifaceted nature of upper limb contributions to soccer kicking performance.

### 2.3. Eligibility Criteria

This review included peer-reviewed research articles, including original research, systematic reviews, and meta-analyses. Studies were included if they investigated biomechanical aspects of kicking or striking movements, examined kinetic chain principles in athletic performance, or explored joint stability, proprioception, and motor control mechanisms. Research examining the effects of external support devices on proprioceptive function or athletic performance was also considered. Only English language publications involving human participants were included in the analysis. Non-peer-reviewed sources were excluded, with the exception of credible gray literature published by recognized academic or governmental institutions. Studies focusing exclusively on injury epidemiology or pathology without direct performance implications were excluded. Research conducted on populations with significant neurological or musculoskeletal pathologies that could confound the interpretation of findings was excluded. Conference abstracts without accompanying full-text publications and studies lacking sufficient methodological detail to enable meaningful synthesis were also excluded from the review.

Because the primary question, whether hand–wrist bandaging could theoretically influence soccer kicking performance, has no corresponding direct basis in the literature, the eligibility criteria were intentionally designed to capture adjacent evidence from mechanistically related domains. These include (a) soccer kicking biomechanics with upper limb components, providing direct evidence on upper limb function in kicking; (b) kinetic chain literature from comparable athletic tasks such as throwing and striking, providing indirect evidence on proximal-to-distal sequencing; (c) proprioception and taping literature from lower-extremity joints, providing indirect evidence on external support mechanisms; and (d) wrist–hand stability and whole-body performance literature, providing indirect evidence on distal upper limb contributions to whole-body coordination. Readers should note that evidence from domains (b), (c), and (d) is indirect and translational: it supports the biological plausibility of mechanisms that have not been tested in the target task or target population. This heterogeneity is a recognized feature of scoping reviews addressing genuinely novel hypotheses, but it substantially limits the inferential strength of any synthesis.

### 2.4. Selection Process

Two reviewers independently screened titles and abstracts against eligibility criteria. Full-text articles of potentially relevant studies were retrieved and assessed independently. Disagreements were resolved through discussion or consultation with a third reviewer. Reference lists of included studies were hand-searched, and forward citation tracking was performed to identify additional relevant literature.

### 2.5. Data Extraction and Analysis

Data were extracted using a standardized template capturing study characteristics, including authors, year, journal, study design, sample size, and population characteristics such as skill level, age, and gender. Methodological details were recorded, encompassing motion capture specifications, EMG protocols, force platforms, and outcome measures. Key findings were documented, including quantitative results, effect sizes, statistical significance, and kinematic or kinetic parameters. Each study was assigned to a primary thematic category, including upper limb biomechanics, kinetic chain principles, proprioception, and external support. Quality indicators were noted, specifically study design rigor, sample representativeness, and measurement validity. One reviewer extracted all data; this represents an acknowledged methodological limitation of the present review, as single-reviewer extraction carries an inherent risk of extraction bias. A second reviewer independently verified accuracy on a random 20% sample, achieving 94% agreement. Readers should interpret extracted data with this limitation in mind, and future reviews on this topic should implement dual independent extraction throughout.

Given the heterogeneous nature of the included studies and the exploratory purpose of scoping reviews, a narrative synthesis approach was employed, with findings organized by thematic categories. The literature was systematically grouped into four primary themes: upper limb biomechanics in soccer kicking, kinetic chain principles and mechanisms, wrist–hand stability and whole-body performance, and proprioceptive enhancement via external support devices. Within each thematic category, the analysis identified consistent findings across multiple studies, examined quantitative parameters and effect magnitudes reported, evaluated methodological approaches and measurement techniques employed, and highlighted gaps and inconsistencies requiring further investigation. Following the thematic analysis, evidence was integrated across all themes to construct a comprehensive biomechanical rationale linking hand–wrist stabilization to potential kicking performance enhancement.

#### Tabular Presentation of Included Studies

In addition to narrative synthesis, a structured summary table was compiled for all 51 included studies [2,3,5,12,13,16,23,24,25,26,27,28,29,30,31,32,33,34,35,36,37,38,39,40,41,42,43,44,45,46,47,48,49,50,51,52,53,54,55,56,57,58,59,60,61,62,63,64,65,66,67]. The table records, for each study: author and year; study design; population (skill level, age, and sex); sport or task; primary outcome; and thematic classification (Table 1).

### 2.6. Quality Assessment

Consistent with scoping review methodology [20,21], we did not perform a formal quality assessment using standardized tools. However, methodological quality was noted during data extraction, with attention to the use of three-dimensional versus two-dimensional motion capture, sample size adequacy, and presence of a control group. Study quality was considered when interpreting findings. Studies with significant methodological limitations were identified during the synthesis process.

## 3. Results

### 3.1. Search Results and Study Selection

The systematic search identified 3847 unique records after duplicate removal. Title and abstract screening excluded 3012 records not meeting the inclusion criteria. Full-text assessment of 189 articles resulted in exclusion of 138 articles, with primary reasons including focus solely on injury without performance implications (n = 52), insufficient biomechanical detail (n = 41), and no upper limb component (n = 27). Reference list screening and citation tracking identified 26 additional relevant articles. The final analysis included 51 studies distributed across thematic categories: upper limb biomechanics in kicking, kinetic chain principles, wrist–hand stability and performance, and proprioception and external support.

**Figure 1:**
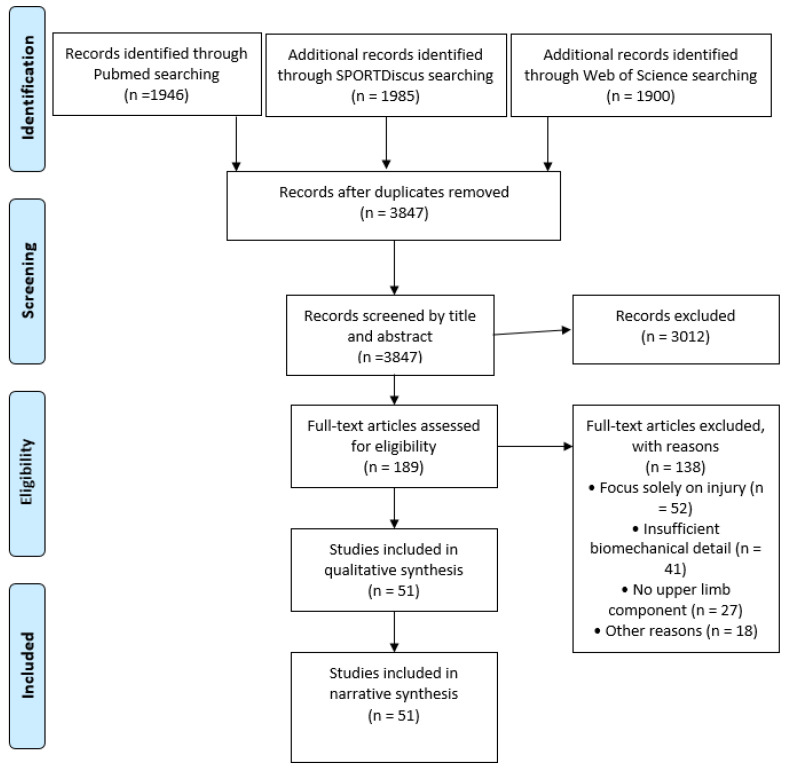
PRISMA-ScR flow diagram. Note: No quantitative synthesis (meta-analysis) was performed in this review. The flow diagram reflects a narrative synthesis approach, consistent with scoping review methodology [20,21]. Any pre-populated template language referring to meta-analysis should be disregarded.

The selection process is documented in a PRISMA-ScR flow diagram (Figure 1).

### 3.2. Study Characteristics

Included studies spanned 1988 to 2025. Publication frequency increased markedly after 2010, with 41 studies (80%) published during this period, reflecting advances in three-dimensional motion capture technology. Studies originated from 16 countries across five continents, with the highest representation from Japan (n = 9), the USA (n = 8), China (n = 6), and European nations (n = 18 combined). Study designs included cross-sectional observational biomechanical studies (n = 31, 61%), narrative or systematic reviews (n = 12, 24%), randomized controlled trials (n = 6, 12%), and case–control comparisons (n = 2, 4%). Sample sizes ranged from 7 to 247 participants, with a median of 18. Skill levels included elite or professional athletes (n = 21 studies), skilled or collegiate athletes (n = 20), recreational participants (n = 7), and mixed populations (n = 3). Gender distribution comprised male-only studies (n = 28), female-only studies (n = 6), and mixed or comparative studies (n = 17). Measurement technologies employed included three-dimensional motion capture systems (n = 25, ranging 100–4500 Hz), surface electromyography (n = 11), force platforms (n = 9), high-speed video analysis (n = 6), and dynamometry (n = 4).

### 3.3. Theme 1: Upper Limb Biomechanics in Soccer Kicking

Shan and Zhang’s [4] comprehensive three-dimensional analysis of male soccer players using multi-marker motion capture introduced the tension arc concept, representing a systematic upper body coordination pattern essential for maximal instep kicks. The tension arc formation phase involves trunk twisting toward the non-kicking side with substantial peak rotation angles, non-kicking side shoulder extension and abduction creating eccentric loading across the trunk and shoulder girdle, and maximum distances between the kicking-side hip and contralateral shoulder. The tension arc release phase coordinates upper trunk flexion and rotation toward the kicking side with leg downswing initiation, creating a whip-like energy release pattern where peak trunk rotation velocity occurs shortly before ball contact [13].

Tension arc magnitude, quantified as the maximum kicking-side hip to non-kicking-side shoulder distance, correlated significantly with ball velocity (r = 0.73, *p* < 0.01), establishing upper body coordination as a determinant of kick quality [4]. Contrary to traditional lower-limb-centric models, biomechanical analyses across multiple studies demonstrate substantial trunk and upper body energy contributions [2,3,4,5,13,23,24,25,26,27,28,29,30,60]. Trunk rotation contributes meaningfully to total ball velocity independently of leg mechanics [24,25,26,27,68]. Shoulder–hip separation angles at ball contact in elite players show larger separations correlating with higher ball velocities [23,60,68]. Non-kicking shoulder displacement during the kick cycle reaches substantial magnitudes [3,4,13,60], indicating considerable upper body movement amplitude. Shinkai et al. [68] confirmed through synchronized high-speed video at 4500 Hz and force transducers that trunk twist magnitude directly affects ball velocity through enhanced energy transfer efficiency in elite Japanese players.

Multiple investigations identify specific functional roles for arm movements during kicking [3,5,13,23,24,25,28,60]. Arms move in opposition to the kicking leg to counteract rotational momentum and maintain dynamic balance during single-leg support [5,13,23,24,25]. The support arm, contralateral to the kicking leg, shows consistent extension during backswing and flexion during forward swing, contributing substantially to balance maintenance calculated from center of mass displacement [3,5,13,28]. Li et al. [69] used continuous relative phase analysis in recreational players to demonstrate that contralateral arm movement initiates the kinetic chain sequence, with energy propagating from the trunk through the shoulder girdle to the kicking leg. Consistent phase angle relationships were documented between trunk and shoulder, shoulder and arm, and trunk and pelvis. The contralateral arm consistently precedes kicking thigh motion [69].

Arm positioning helps stabilize the center of mass trajectory during the single-leg support phase, optimizing conditions for force application by the kicking leg [3,5,13,23,24,28]. Support arm position variance is significantly lower during accurate kicks compared to inaccurate kicks [29,70], suggesting proprioceptive awareness of arm position influences kick precision [29,30]. Elite players demonstrate superior upper-body coordination compared to non-elite players across multiple parameters [14]. Trunk rotation range of motion, arm movement amplitude, trunk rotation timing consistency, and ball velocity all show substantial differences between skill levels. These differences suggest that upper-body coordination is trainable and distinguishes between skill levels.

Female players utilize different upper-body coordination strategies compared to males [60,61]. Greater trunk flexion, more extensive arm extension, and earlier and more sustained erector spinae activation characterize female players [71]. These differences likely reflect biomechanical and neuromuscular adaptations that achieve similar functional outcomes through alternative coordination solutions [29]. Available evidence from multiple primary studies indicates that upper limbs play a substantial and functionally relevant role in optimal kicking performance, though this conclusion is based on observational biomechanical data rather than controlled experimental manipulation. Lees et al.’s [5] seminal review of over 100 studies concluded that future research should employ full-body models to adequately capture upper–lower body integration.

### 3.4. Theme 2: Kinetic Chain Principles and Bidirectional Influences

Kibler et al. [10] formally defined core stability as the ability to control the position and motion of the trunk over the pelvis to allow optimum production, transfer and control of force and motion to the terminal segment in integrated athletic activities. This definition explicitly includes terminal segments, specifically the hands, as recipients of transferred force and motion, providing theoretical justification for investigating distal upper limb influences on integrated athletic performance. The fundamental kinetic chain principle posits that proximal segments provide stable bases enabling efficient force generation and transfer to distal segments [10,72]. This creates conditions for optimal timing of distal segment movements, enhanced positioning accuracy, maximal velocity generation at endpoints, and reduced injury risk through load distribution.

Biomechanical analysis of taekwondo roundhouse kicks in elite athletes using high-speed motion capture revealed clear velocity progression demonstrating summation of speed [73]. Progressive increases from thigh to shank to foot velocities were documented. Each distal segment began acceleration as the proximal segment reached peak velocity, with consistent inter-segment timing intervals [12,31,32,36,61]. This sequential coordination exemplifies Putnam’s [9] theoretical principle that optimal timing occurs when proximal segment peak velocity coincides with distal segment acceleration onset.

Kim et al. [74] quantified joint work contributions in kicking using inverse dynamics, revealing that the hip contributes substantially as the primary energy generator, the knee contributes moderately as a postural stabilizer, the ankle provides considerable contribution through spring-like energy storage and return, and toe joints contribute to fine-tuning. This distribution confirms that proximal joints generate the foundation for distal velocity while distal joints refine and transmit forces [12,31,32,36]. Hirashima et al.’s [61] electromyographic study documented the proximal-to-distal muscle activation sequence in overarm throwing, revealing that even upper limb movements begin with lower-body activation. Systematic progression occurs from contralateral gastrocnemius and soleus, through ipsilateral gluteus maximus, external oblique, latissimus dorsi, pectoralis major, triceps brachii, and finally wrist flexors, all occurring before ball release. Timing precision was critical, as variance in inter-muscle timing intervals correlated negatively with ball velocity [61], demonstrating that coordination quality, not just muscle strength, determines performance.

Synthesis across striking and throwing sports demonstrates consistent patterns of energy distribution [7,12,31,32,33,35,37,38,68]. Legs and trunk contribute the majority of total kinetic energy [34,37], the shoulder complex provides moderate contributions [34,38,68,75,76,77], and elbow–wrist segments account for the remaining contributions [31,33,35]. Proximal segment deficits are associated with substantially increased distal compensatory demands, as quantified in kinematic and kinetic analyses of striking sports [7]. Almansoof et al.’s [7] comprehensive review quantified critical relationships showing that when proximal energy contribution decreases substantially, distal segments must compensate with considerably increased velocities to maintain performance, resulting in markedly greater joint stress at compensating segments. This has profound injury implications, as substantial proportions of throwing shoulder injuries demonstrate identifiable trunk and lower limb deficits upon clinical examination [31,78].

Traditional kinetic chain models emphasized unidirectional force flow from proximal to distal segments [9]. However, recent research demonstrates bidirectional influences [7,15]. Terminal segment positioning affects proximal segment coordination through mechanical coupling and proprioceptive feedback. Studies on passive trunk stabilization demonstrate that proximal stability enhances distal hand control and grip strength [33,35,79], while grip strength predicts postural balance performance [80], suggesting reciprocal relationships. The implications of ankle bandaging for cross-body sensorimotor effects, and the limitations of available evidence in this regard, are examined in Section 3.6 [62]. These findings, taken together, are theoretically consistent with the concept of bidirectional kinetic chain influences; however, the evidence base for bidirectionality remains predominantly indirect, derived from clinical and non-soccer populations, and should not be interpreted as established fact in the context of healthy elite soccer athletes. The mechanistic pathways proposed here require direct experimental testing.

Kibler et al. [10] described the thoracolumbar fascia as an integrated force transmission system connecting lower limbs via gluteus maximus attachments to upper limbs via latissimus dorsi attachments, forming a stabilizing ring around the abdomen. This fascial system provides structural continuity for force transfer between the upper and lower body, supporting the biomechanical plausibility of bidirectional kinetic chain influences [12,36].

### 3.5. Theme 3: Wrist–Hand Stability and Whole-Body Performance

Lee et al. [80] investigated the effects of wrist position and stabilization on maximal grip force in healthy adults, finding that wrist position significantly affected grip force capacity. The multiarticular arrangement of finger flexor tendons means wrist position creates length–tension changes affecting grip capacity through biomechanical constraints, and external stabilization optimizes these length–tension relationships. Ambike et al. [81] examined how isometric hand grip force affects wrist kinematics during mechanical perturbations. The findings demonstrate that hand and wrist function represent an integrated neuromuscular control system rather than independent joints, suggesting interventions targeting either component may influence the entire distal upper extremity.

A critical review synthesizing multiple studies established moderate-to-strong correlations between core stability test performance and upper limb athletic performance metrics [10,82,83,84]. Empirical studies in collegiate athletes confirmed specific relationships [83,84]. Y-balance lower quarter composite score correlated with upper extremity closed kinetic chain test performance. Plank hold time correlated with medicine ball throw distance. Side plank endurance correlated with unilateral shot put distance. Proposed mechanisms include: a stable proximal base enabling distal precision and power; the core providing a kinesthetic reference frame for limb position sense; and trunk muscle activation patterns influencing limb motor control via fascial connections [10]. Core training programs improve upper extremity performance substantially even without direct upper limb training [10,82,83,84], demonstrating that proximal optimization enhances distal function.

Gagnon et al. [78] examined how passive trunk stabilization influences hand grip strength during manual wheelchair propulsion tests. Kinematic analysis showed trunk stabilization reduced compensatory shoulder movements, suggesting more efficient force transmission through the kinetic chain [78]. These findings support the proximal-to-distal principle while suggesting the inverse may also hold: if proximal stability enhances distal control, might distal stability enhance proximal-to-distal coordination? The relationship between grip strength and postural balance has been explored in observational studies, with some evidence suggesting that hand strength may correlate with whole-body postural control metrics [79]. Proposed mechanisms include grip strength as a proxy for overall neuromuscular function, hand proprioceptive input influencing postural control systems, and shared neural pathways for hand and postural control.

An intervention study demonstrated that upper limb training in standing positions improves postural control in healthy adults [85]. A four-week program of arm-reaching tasks while standing reduced postural sway, improved limits of stability, and enhanced dynamic balance scores. Critically, transfer effects were demonstrated as arm training produced improvements in balance tasks not involving arms [85]. Results support bidirectional upper limb–whole body coordination, suggesting that optimizing arm function, including distal stability, may enhance whole-body motor control. Hong et al. [64,85] showed that grip-strengthening exercises combined with wrist stability training constitute an effective intervention for improving pain, function, grip strength, and muscle strength, emphasizing the need for wrist exercise interventions in patients with non-specific chronic wrist pain.

Additionally, recent meta-analytic evidence from therapeutic contexts demonstrates that hand-focused strength and proprioceptive training improves grip strength with moderate effect sizes, with particularly strong improvements in elderly populations and substantial cross-sectional benefits on manual motor skills [84,86]. These findings suggest that sensory input and hand–wrist stability modulate common central motor circuits, with possible repercussions on balance and intersegmental coordination even in complex whole-body movements. Studies on elite athletes show that interventions such as contractions of the non-dominant hand alone improve motor performance under high-pressure conditions [86], clarifying how the sensory and efferent components of the hand–wrist segment can act as neurofunctional priming mechanisms in managing coordination tasks. Furthermore, research on hand–eye laterality highlights increased prevalence of crossed laterality profiles among elite athletes in sports such as soccer, golf and tennis [87], suggesting that interaction between dominant hand and visual–motor function contributes to greater executive efficiency in managing technical and anticipatory-kinematic components of movement. This asymmetry is attributed to possible functional superiority in tasks with high neuromotor demand, though mechanisms remain partly indirect.

### 3.6. Theme 4: Proprioceptive Enhancement via External Support

Riemann and Lephart [18] established the theoretical framework for proprioception’s role in functional joint stability, defining proprioception as a specialized variation in touch encompassing joint position sense representing static awareness of joint angles, kinesthesia representing dynamic awareness of movement and velocity, and sense of force and tension representing perception of muscle force magnitude. Proprioceptive information arises from diverse mechanoreceptors [18]. Joint capsules contain Ruffini endings that are slow-adapting and respond to position and velocity, Pacinian corpuscles that are rapid-adapting and respond to acceleration, and Golgi-like endings that respond to stress and strain. Muscles contain muscle spindles, responding to length and velocity, and Golgi tendon organs, responding to tension. The skin contains Meissner’s and Merkel’s corpuscles responding to stretch and deformation. Afferent pathways project to the spinal cord for reflex loops, the cerebellum for coordination, and the cortex for conscious awareness, guiding muscle activation through feedforward anticipatory and feedback reactive mechanisms.

Ghai et al.’s [16] 2024 systematic review examined taping effects on lower extremity proprioception, searching five databases through December 2023. The overwhelming majority of included studies demonstrated significant proprioceptive enhancement with taping. Effect magnitudes demonstrated substantial reductions in joint position sense error, representing moderate-to-large effects. Kinesio tape produced moderate effects, rigid tape produced larger effects, and joint position sense error reduction ranged substantially across studies. Proposed mechanisms [16] include increased cutaneous mechanoreceptor stimulation from skin stretch during movement, enhanced alpha motor neuron pool excitability via sensory input, improved cortico-spinal pathway facilitation, and heightened conscious awareness of joint position through attention mechanisms. Effects occur immediately upon application and persist throughout wear duration, suggesting primarily sensory rather than training-mediated mechanisms.

A systematic review and meta-analysis by Ghai et al. [65] examined taping effects on force sense accuracy across 11 studies involving 279 participants. Results demonstrated improved force matching accuracy with taping compared to no taping and compared to placebo tape, with statistically significant improvements in both absolute and relative force sense accuracy. Effects were consistent across joints, including the ankle, knee, wrist, and elbow and across tape types [16,39,40,41,42,43,44,45,66,67]. These findings are directly relevant to athletic performance requiring precise force application, such as kicking, where force magnitude and timing critically determine ball velocity and accuracy.

Henderson et al. [17] established that external ankle support produces measurable neuromuscular effects beyond simple mechanical restriction. Effects were attributed to increased afferent input from cutaneous and joint mechanoreceptors stimulated by brace pressure and skin stretch during movement. External ankle support in individuals with chronic ankle instability has been shown to modify gait kinematics, with elastic and athletic taping producing different kinematic profiles during walking [67]. These findings suggest that external joint supports can alter movement mechanics. Heß et al. [62] conducted a within-subjects crossover RCT examining the immediate effects of ankle bandaging on fine coordination, proprioception, balance, and gait in 70 patients with subacute ankle sprains. The bandage produced significant improvements in single-leg balance and gait parameters. However, no significant improvement was observed in ankle-specific fine coordination or proprioception, and upper limb outcomes were not assessed at any stage of the study. Consequently, this study provides no empirical evidence, in either direction, for cross-body sensorimotor effects of distal external support on remote body segments. The neurophysiological plausibility of such effects must instead be derived from established theoretical frameworks on proprioceptive contributions to central motor planning [18,88], from basic wrist mechanoreceptor science [55,58], and from the documented role of afferent input in inter-joint coordination [88], rather than from direct experimental precedent. This represents the most speculative component of the proposed theoretical framework, and its empirical status must be explicitly recognized as unresolved.

Sainburg et al. [88] examined the effects of unilateral deafferentation, representing proprioception loss due to sensory neuropathy, on inter-joint coordination in participants with matched controls. Deafferented participants showed substantially increased coordination timing errors, increased movement variability, and markedly reduced joint coupling measures. Critically, coordination deficits appeared bilaterally, not just in the affected limb, suggesting that proprioception contributes to central motor planning processes [88]. These results confirm that proprioception is necessary for precise inter-joint coordination. By inference, proprioceptive enhancement via external support should improve coordination quality.

Salva-Coll et al. [89] confirmed that sophisticated mechanoreceptor systems exist in wrist joints, providing precise proprioceptive information essential for motor control [90,91]. Marchal-Crespo et al. [92] examined how proprioceptive augmentation via robotic haptic guidance improves movement quality in participants performing arm reaching tasks. Additional proprioceptive feedback produced substantial movement error reduction, kinematic variability reduction, and accelerated learning rates. Effects persisted after feedback removal, indicating genuine motor learning. These findings suggest that enhanced sensory feedback, whether from robotic devices or external supports like bandaging, can improve movement quality and accelerate skill acquisition in sports contexts.

The literature on electromyography, proprioception and muscle fatigue suggests that muscle redundancy in the hand–wrist unit guarantees an adaptive reserve that reduces the risk of motor impairment even after intense stress, a typical scenario in elite competition [93,94]. The bandage could, in this perspective, enhance skin-articular proprioception and stabilize upper limb kinematics during running and kicking dynamics, promoting neuromuscular anchoring useful in force production, precision and postural balance.

Confirmed enhancement mechanisms [19] include cutaneous mechanoreceptor stimulation increasing afferent traffic to the central nervous system, skin stretch activating slowly adapting receptors to provide continuous joint position feedback, external pressure enhancing joint capsule mechanoreceptor sensitivity, improved conscious body schema representation, and enhanced motor planning through better sensory predictions. Strong consensus emerged that proprioceptive enhancement strategies improve functional performance and reduce injury risk across multiple sports contexts.

For the wrist to function as a proprioceptive organ capable of influencing distal motor output, the anatomical substrate must first be established. Immunohistochemical studies have confirmed that wrist ligaments contain both Ruffini-like mechanoreceptors, which encode sustained tensile strain and joint position, and Paciniform corpuscles, which respond to rapid dynamic perturbations, in densities that vary systematically between ligaments, with the dorsal intercarpal, dorsal radiocarpal, and scapholunate interosseous ligaments being particularly richly innervated [90,91]. Neurophysiological studies have demonstrated the importance of joint-protective ligamento-muscular reflexes, whereby a polysynaptic reflex arc originating from ligamentous and capsular sensory nerve endings influences the activity of joint-stabilizing musculature via gamma-motoneurons, thereby coordinating muscle tone to maintain joint stability during both rest and movement [7]. Wrist joint proprioception must therefore be understood as part of a sensorimotor system in which afferent information from nerve endings in the wrist joint affects the neuromuscular control of the joint [5]. External compression applied by a wrist bandage against the dorsal capsule, overlying skin, and subcutaneous tissue is hypothesized to augment mechanoreceptor discharge within this system, a mechanism analogous to the skin-stretch-mediated enhancement of proprioceptive acuity documented at other joints [55,58], though whether such augmentation produces functionally significant changes in upper limb motor control during high-velocity athletic movements such as the soccer instep kick remains, at present, an untested hypothesis requiring direct empirical investigation.

In summary, although no specific randomized controlled trials or meta-analyses are available on the use of hand–wrist bandages in elite soccer performance, the collected systematic reviews and empirical evidence support a neurophysiological and biomechanical rationale that the upper limb, through proprioception, muscle stability, and sensory feedback, directly contributes to lower limb motor control and the quality of technical gestures such as kicking [16,18,45,46,47,48,55,58,65]. The collected evidence provides theoretical, mechanistically plausible support for the hypothesis that the upper limb, through proprioception, neuromuscular stability, and sensory feedback, may contribute to lower limb motor control and kicking quality. However, it is essential to clarify that no study in this review directly tests hand–wrist bandaging in soccer players, and the evidence chains from adjacent domains involve substantial inferential steps across different joints, populations, and performance contexts. These data support the hypothesis as worthy of direct empirical investigation; they do not, at present, justify clinical or practical recommendations for its use.

### 3.7. Integrated Synthesis and Proposed Mechanisms

Synthesizing evidence across the four themes reveals three converging lines of evidence supporting the theoretical plausibility that hand–wrist bandaging could enhance soccer kicking performance. First, upper limbs are biomechanically essential for optimal kicking through tension arc formation, counterbalancing, kinetic chain initiation, and energy transfer coordination, with elite players demonstrating superior upper body coordination, distinguishing them from novices. Second, available evidence is consistent with bidirectional kinetic chain operation: proximal stability demonstrably enhances distal control, and observational associations between distal upper limb function and whole-body postural metrics suggest possible reciprocal influences. However, direct cross-body sensorimotor effects, specifically, whether proprioceptive input from the hand–wrist region can influence lower-extremity motor control during kicking, remain theoretically plausible on neurophysiological grounds [18,88] but have not been empirically demonstrated in any athletic population included in this review. Third, external joint support at lower-extremity joints has been shown to enhance proprioception and force perception through increased mechanoreceptor stimulation, with moderate effect sizes documented in systematic reviews [16] and meta-analyses of force sense accuracy [65]. Whether such effects extend across body regions, specifically from the distal upper extremity to the lower extremity in the context of kicking, remains theoretically plausible on neurophysiological grounds [18,19,55,88] but has not been directly tested in any athletic population. No study included in this review was designed to detect or measure cross-body sensorimotor effects in the direction relevant to the present hypothesis; the absence of such evidence is itself a critical finding of this scoping review and defines the primary research gap. The cross-body sensorimotor hypothesis, while conceptually coherent, must be regarded as untested.

Based on synthesized indirect evidence, we propose three mechanistically distinct pathways through which hand–wrist bandaging could theoretically influence kicking performance: Mechanism 1: Enhanced sensorimotor afference: Hand–wrist bandaging increases cutaneous mechanoreceptor stimulation [16,18], providing enriched afferent feedback about upper limb position, movement velocity, and acceleration during arm swing and tension arc formation. Enhanced proprioceptive acuity may improve precision and consistency of upper body contributions to the kinetic chain, consistent with documented reductions in joint position sense error with taping [16] and improvements in force perception accuracy [65].

Mechanism 2: Kinetic chain optimization: Improved distal upper limb stability provides a more reliable proprioceptive reference point for proximal segment coordination, potentially reducing arm positioning variability during the balance-critical single-leg support phase and enhancing force transmission efficiency from trunk through shoulder girdle. This is consistent with the principle that proximal deficits increase compensatory demands at distal segments [7] and with evidence that trunk stabilization enhances distal hand control [82].

Mechanism 3: Whole-body neuromechanical coordination: Cross-body sensorimotor effects are theoretically plausible on the basis of established neurophysiological frameworks describing proprioceptive contributions to central motor planning [18,88] and on wrist mechanoreceptor science [55,58], but have not been directly demonstrated in any athletic population in the reviewed literature. Hand–wrist bandaging may theoretically modulate body schema representation, central pattern generator function for complex multi-limb movements, and interhemispheric coordination through enriched afferent input; however, this remains the most speculative of the three proposed mechanisms and requires the most direct empirical investigation. A fourth potential factor, psychological confidence, has been mentioned in prior literature on external support devices. While subjective confidence may interact with biomechanical mechanisms, this effect is not directly supported by data in the present context and is therefore considered a secondary, speculative consideration rather than a primary mechanism.

## 4. Discussion

### 4.1. Summary of Key Evidence

The evidence base supporting this theoretical hypothesis rests on three domains of indirect evidence, detailed in the Results Section: (1) substantial upper limb biomechanical contribution to soccer kicking [3,5,13,23,24,28,29,30,60]; (2) bidirectional operation of kinetic chains [7,10,12,15,36,37,38]; and (3) demonstrated proprioceptive enhancement via external joint support [16,18,19,39,43,44,45,46,65,66]. Rather than re-enumerating specific findings already presented in the Results Section, the following sections address the interpretive weight of this evidence, its limitations, and its implications for research and practice.

### 4.2. Critical Research Gap

Despite strong theoretical foundations and supporting evidence from related domains, direct empirical testing of hand–wrist stabilization effects on kicking performance is completely absent from the literature. This represents the most significant gap and highest research priority. The hypothesis remains untested for several potential reasons. Traditional research paradigms focus predominantly on lower limb mechanics in kicking. Assumptions persist regarding minimal distal upper limb contribution to lower limb tasks. The lack of conceptual frameworks linking distal upper extremity to lower extremity performance indicates limited investigation in this area. Methodological challenges exist in isolating subtle coordination effects. The novelty of bidirectional kinetic chain perspectives emerged primarily after 2015, leaving insufficient time for comprehensive investigation.

### 4.3. Theoretical Plausibility Assessment

The rationale has several strengths. The theoretical plausibility of the hypothesis is supported by the convergence of indirect evidence across the three domains outlined above. It must be explicitly acknowledged that no study identified in this scoping review directly demonstrates that external support at one body region produces measurable effects on motor control of a distant body region in healthy athletes. As detailed in Section 3.6, the only potentially relevant study [62] found no cross-body sensorimotor effect and did not assess upper limb outcomes; this pillar of the proposed rationale therefore remains entirely hypothetical. The available biomechanical evidence from multiple three-dimensional motion capture studies indicates substantial upper limb contribution to kicking performance [14]. With respect to cross-body sensorimotor effects, the scoping review identified no study that directly demonstrated that external support at one body region produces measurable effects on motor control of a distant body region in healthy athletes. Heß et al. [62] examined ankle bandaging in patients with subacute ankle sprains and found improvements in balance and gait but no significant effect on the ankle fine-coordination outcome measures. Consequently, this pillar of the proposed rationale remains hypothetical rather than empirically supported, and must be explicitly recognized as the weakest component of the theoretical framework presented here.

Potential limitations exist as well. Ceiling effects in elites may occur where elite players already demonstrate optimal upper-body coordination [14]. Proprioceptive enhancement magnitude may be insufficient to produce measurable performance improvements at the highest skill levels, given the modest effect sizes documented for joint position sense improvement with taping [16,65], the predominance of null performance results reported across athletic populations [49], and the absence of functional performance improvements with taping specifically in healthy soccer players [48]. Individual variability exists where large inter-individual differences in upper body coordination strategies [70,71] suggest effects may not be universal, and responders versus non-responders could emerge. Magnitude questions remain, where, while proprioceptive enhancement is proven with meaningful joint position sense error reduction [16], whether this translates to meaningful kicking performance improvements remains unknown. Mechanical restriction concerns exist where overly tight bandaging could mechanically restrict wrist movement, potentially impairing rather than enhancing function, and optimal tension balance requires empirical determination.

### 4.4. Skill Level Considerations

Effect magnitudes likely vary by skill level. Elite players already demonstrate near-optimal upper body coordination with minimal timing variability [14]. Effects might manifest as subtle consistency improvements rather than magnitude enhancements, including reduced performance variability across multiple kicks, maintenance of coordination quality under fatigue or pressure, and enhanced psychological confidence in arm positioning[95,96]. Developing players may show larger benefits as proprioceptive enhancement accelerates learning of proper coordination patterns through faster acquisition of tension arc timing; more rapid development of arm–trunk–leg coupling; reduced reliance on conscious control, thus facilitating the automatization of movement patterns; and potential utility as a training tool rather than as an acute performance aid. Recreational players might demonstrate the largest absolute effects given greater baseline coordination variability, though practical implications are less significant than for elite populations.

### 4.5. Gender-Specific Considerations

Given distinct upper body strategies between males, who demonstrate greater trunk rotation and less arm extension, and females, who show greater trunk flexion and more arm extension [4,26,27,60,97], effects may manifest differently. For male players, bandaging might enhance rotational coordination precision and tension arc consistency, given the reliance on trunk rotation velocity [4,25,26]. For female players, effects might manifest more in stabilization during the flexion-dominant phase and fine motor control of arm extension amplitude [60,71,97,98]. Gender-comparative studies are essential for the comprehensive understanding of how proprioceptive enhancement interacts with different movement strategies employed across biological sexes [16,49,97].

### 4.6. Contextual Factors and Moderators

Several contextual factors may moderate effect magnitudes. Effects might differ meaningfully across kick types [3,5]. Maximal instep kicks for power shots depend critically on tension arc quality and whole-body coordination [4,5,24]. Inside foot passes requiring precision depend substantially on fine motor control and postural stability [3,28,29]. Volleys and aerial techniques depend heavily on balance maintenance during challenging body positions [5,13]. Weak foot kicks may show potentially larger effects given less automatized coordination patterns and greater reliance on conscious control mechanisms [14,28].

Game situation factors include contested kicks requiring rapid adjustment, where proprioceptive enhancement may provide greater advantage through improved reactive coordination [16,18,19], uncontested set pieces where effects might be minimal given the opportunity for optimal preparation and reduced time pressure, and fatigue states where proprioceptive acuity naturally declines with physical exhaustion and bandaging might attenuate this decline [30,44,55]. Individual anatomy factors include arm length and trunk flexibility affecting upper body contribution patterns [4]; players with naturally superior proprioception may show minimal benefit due to ceiling effects [14,16], and those with proprioceptive deficits from prior injuries might respond more substantially to augmented sensory input [18,19,66].

### 4.7. Implications for Other Sports

If validated in soccer, similar principles might apply to martial arts kicking, including taekwondo, karate, and kickboxing, where upper body coordination is recognized but distal stability remains unexplored [73,74]. Rugby kicking, involving place kicking and punting, shows similar whole-body coordination patterns with upper limbs contributing to balance and force generation [99]. Volleyball spiking represents overhead striking with significant trunk and arm coordination requirements, where hand–wrist stability might enhance control and power [100]. Tennis serving demonstrates a clear kinetic chain from the legs through the trunk to the arm with the hand–wrist as a critical terminal segment affecting ball control [8,34]. Baseball and softball pitching show overhead throwing with a well-documented kinetic chain where hand–wrist stability might enhance both velocity and accuracy [31,32,33,35].

The hypothesis represents a potentially generalizable principle: optimizing terminal segment stability may enhance proximal-to-distal coordination efficiency in any integrated kinetic chain movement. This principle extends beyond kicking to virtually any whole-body athletic skill requiring coordinated multi-segment sequencing and precise timing relationships between body regions.

### 4.8. Practical Implementation Considerations

Bandaging specifications that would need to be systematically evaluated in future research include: optimal coverage area (wrist only versus wrist–hand versus forearm–wrist–hand); tension level sufficient for proprioceptive stimulation without mechanical restriction; material properties (elastic kinesio tape, rigid athletic tape, or compression bandage); and unilateral versus bilateral application. These parameters cannot be determined from existing evidence and must be empirically optimized before any recommendations, even provisional ones, can be made to practitioners or athletes.

Given the complete absence of direct evidence, it would be premature to recommend hand–wrist bandaging as a performance intervention for elite soccer players, even on an exploratory or cautious basis. The appropriate next step is controlled laboratory research as described in Section 4.10, not field implementation. Practitioners who choose to experiment with this approach in the interim do so entirely without evidence-based justification, and systematic documentation of outcomes, while potentially informative, does not substitute for rigorous controlled research.

### 4.9. Limitations

Methodological limitations exist in this scoping review. The scoping review design involves no formal quality assessment using standardized tools such as PEDro or Cochrane Risk of Bias, which is consistent with scoping review methodology [20,21]. However, this limits the strength of evidence synthesis and precludes definitive conclusions about effect reliability. Furthermore, no statistical meta-analysis was performed, given the heterogeneous outcomes and study designs across the included articles. Narrative synthesis is inherently subject to interpretation bias despite systematic data extraction procedures, though multiple reviewers mitigated but did not eliminate subjectivity in thematic categorization and evidence integration.

Publication bias may have introduced a systematic tendency toward positive findings in the proprioception and taping literature, though included systematic reviews showed high proportions of positive effects [16,65], suggesting genuine effects rather than publication bias alone, but unpublished negative findings cannot be ruled out. Evidence base limitations exist as well. The absence of direct studies represents the core limitation acknowledged throughout this review, as the hypothesis remains completely untested, with all evidence being indirect or extrapolated from related domains and adjacent research areas.

Cross-joint extrapolation is required, as evidence from ankle and knee taping is applied to wrist applications, though mechanoreceptor mechanisms should theoretically be similar across joints given comparable peripheral nervous system architecture [18]. Sport generalization concerns arise as some kinetic chain evidence derives from throwing and striking sports other than soccer, though fundamental biomechanical principles of sequential coordination should generalize across movement patterns. Elite population underrepresentation exists where many included studies examined skilled or recreational rather than truly elite athletes, limiting the direct applicability to professional soccer contexts where performance optimization is most critical.

Theoretical limitations persist as well. Mechanism speculation remains as proposed mechanisms remain entirely speculative until empirically tested through controlled experimentation, and alternative explanations exist for each mechanism that cannot be ruled out based on current evidence. An unknown effect magnitude exists, where whether proprioceptive enhancement of demonstrated magnitude translates to meaningful kicking performance improvements remains entirely uncertain without direct testing. Possible null effects exist where elite athletes may already optimize available biomechanical and proprioceptive resources through years of intensive training, leaving minimal room for external enhancement interventions.

### 4.10. Future Research Directions

The immediate research priority is a proof-of-concept study employing a within-subjects crossover randomized controlled trial design. The study sample should include elite or highly skilled soccer players with an adequate sample size for statistical power. Conditions should include bilateral wrist bandaging, contralateral or non-kicking-side wrist only, ipsilateral or kicking-side wrist only, and no bandaging as a control condition. Primary outcomes should measure ball velocity using calibrated measurement technology, kick consistency measured as a velocity coefficient of variation across multiple trials, and accuracy measured as target-hitting percentage or deviation from intended target.

Secondary outcomes should include comprehensive three-dimensional kinematics with multiple high-speed cameras capturing trunk rotation parameters, arm positioning throughout the kick cycle, shoulder–hip separation angles, and joint angular velocities across all relevant segments. Surface electromyography should measure trunk muscles, including external oblique and erector spinae and shoulder muscles, including the anterior deltoid and latissimus dorsi, to assess activation timing and magnitude. Ground reaction forces during planted leg contact should be recorded to assess stability and force generation. Subjective ratings should assess comfort levels, confidence in movement execution, and perceived coordination quality. Duration should involve a single session with a counterbalanced condition order and adequate rest between conditions to prevent fatigue confounding.

Analysis should use repeated-measures statistical models with appropriate post hoc pairwise comparisons, effect size calculation using standardized metrics, and responder analysis examining individual differences to identify characteristics predicting benefit. A mechanism isolation study should aim to isolate proprioceptive enhancement from mechanical restriction effects. Design should include multiple conditions: a standard elastic bandage providing proprioception plus mechanical support, a sham bandage with loose application providing minimal proprioception and no restriction, anesthetic cream under a bandage providing mechanical support with blocked proprioception, and no bandaging control. Outcome analysis should determine whether effects are proprioceptively mediated or mechanically driven through differential responses across conditions.

A dose–response study should identify optimal bandaging tension levels. Design should involve multiple bandaging tightness levels ranging from minimal to maximal comfortable tension. Outcomes should include proprioception tests measuring joint position sense and threshold to detection of passive motion, grip strength and force control precision, and kicking performance metrics across all conditions. Analysis should identify tension level maximizing proprioceptive enhancement without mechanical impairment through quadratic or piecewise regression modeling.

A longitudinal training study should examine whether training with bandaging accelerates skill development over time. Design should involve a parallel-group randomized controlled trial over an extended training period with groups including standard kicking training, training with wrist bandaging throughout sessions, and training with proprioceptive focus without bandaging as attention control. Outcomes should measure skill acquisition rate through repeated assessment, retention after intervention cessation to assess learning versus temporary performance enhancement, and transfer to competition settings through match performance analysis. The population should include youth or developing players, given greater learning potential and less optimized baseline coordination.

A sex-comparative study should examine whether effects differ by biological sex, given distinct upper body strategies documented in the literature. Design should use factorial arrangement with gender by bandaging condition, allowing for interaction testing. The analysis should examine gender by condition interaction effects on all performance outcomes to determine whether sex-specific recommendations are warranted. An ecological validity study should translate laboratory findings to field conditions through an observational field study during actual training and competitive matches. Outcomes should include performance statistics such as shot velocity and accuracy from match analysis software, subjective player reports through structured interviews, and coach observations using standardized assessment tools. Technology should employ wearable sensors for ecological measurement that maintain validity while minimizing interference with natural performance.

### 4.11. Methodological Recommendations for Future Research

Biomechanical measurement requirements include the use of full-body models capturing sufficient upper limb detail to assess subtle coordination changes [5]. High-speed motion capture at adequate sampling rates for rapid arm movements, with proper synchronization with force platforms and electromyography systems, should be used to ensure temporal alignment. Multiple kick trials should be collected to assess consistency as a primary dependent variable, given substantial trial-to-trial variability in complex motor skills.

Outcome selection should prioritize ball velocity using calibrated measurement, accuracy using standardized targets, and consistency using appropriate variability metrics as primary outcomes reflecting performance quality. Secondary outcomes should measure kinematics, including joint angles, angular velocities, and segment coordination metrics; kinetics, including ground reaction forces and joint moments if inverse dynamics are employed; and electromyography of relevant trunk, shoulder, and arm muscles. Tertiary outcomes should include proprioception tests independent of kicking performance, subjective ratings of multiple dimensions, and fatigue resistance through repeated measurement blocks.

Within-subjects designs should be employed to maximize statistical power given substantial individual variability in coordination patterns. Mixed-effects models should account for repeated measures structure and individual random effects. Responder analysis should identify individual characteristics predicting benefit magnitude to guide personalized applications. Adequate washout period between conditions should be incorporated if a crossover design is used to prevent carryover effects. Pre-registration and a detailed statistical analysis plan should minimize bias through a priori specification of analyses.

Regarding sample size, investigators should conduct a formal a priori power calculation, informed by documented effect sizes for joint position sense improvement with taping using effect size estimates from the most methodologically comparable studies available. Given the uncertainty about whether equivalent effects are present at the wrist in healthy elite athletes, a population and joint not directly represented in the existing taping–proprioception literature, a conservative effect size assumption and a within-subjects crossover design are recommended to maximize statistical power. Larger samples are necessary for between-subjects comparisons or subgroup analyses, given the reduced statistical efficiency and increased variability.

### 4.12. Implications for Sports Medicine and Performance Science

This hypothesis is consistent with an emerging systems-based perspective in sports biomechanics [7,10,12], according to which interventions targeting one body region may, through proprioceptive and neuromechanical pathways [18,88], influence motor control of distant segments and overall movement quality. Whether such propagation occurs specifically in the context of hand–wrist bandaging and soccer kicking performance remains entirely untested and represents the core empirical gap identified by this review. Bandaging represents an exceptionally low-risk intervention with minimal cost; no adverse effects reported in the extensive proprioception literature [16,65]; easy application requiring minimal training or expertise; reversibility, allowing for immediate removal if the intervention proves ineffective or causes discomfort; and compatibility with existing equipment and training protocols without substantial modifications.

The complete absence of direct empirical evidence in this specific context precludes evidence-based recommendations for clinical or performance use at this stage. Any practitioner interest in this approach should be pursued exclusively within structured, ethically approved research protocols, with outcomes systematically documented to advance the collective evidence base. In elite sport, where performance differences separating competitors are often minimal, marginal gains from multiple small optimizations may accumulate to confer competitive advantage [86]. If hand–wrist bandaging improves ball velocity by even small percentages, this could meaningfully affect shooting success rates, given goalkeeper reaction time constraints and goal mouth dimensions that create narrow performance margins.

A theoretically interesting but entirely untested question is whether wrist proprioceptive feedback during training, assuming it is demonstrably altered by bandaging, which itself remains unestablished, could interact with sensorimotor learning processes [101,102]. The potential relevance of developmental neuroplasticity to such hypothetical effects represents a secondary hypothesis that is premature to evaluate in the absence of any primary evidence for bandaging effects on proprioception or performance in the soccer context. These questions are identified as areas for future investigation, not as an established rationale for current practice.

Furthermore, emerging evidence suggests that hand-focused proprioceptive training produces substantial improvements in grip strength and manual dexterity, particularly in elderly populations, with moderate effect sizes and cross-sectional benefits on motor skill [85]. These findings indicate that sensory input and hand–wrist stability modulate common central motor circuits, with potential repercussions on balance and intersegmental coordination even in complex whole-body gestures. Evidence from motor priming [86] and laterality research [87] (detailed in Section 3.5) further supports the view that hand–wrist sensorimotor function participates in higher-order neural regulation of complex athletic performance, though direct experimental confirmation in soccer is absent. This asymmetry has been attributed to possible functional superiority in tasks with high neuromotor demand, though the underlying mechanisms remain only partially understood.

The fatigue-resistance implications of this evidence base are discussed in Section 3.6.

### 4.13. Limitations of the Hypothesis

Theoretical limitations persist, wherein an unknown effect magnitude means that the proprioceptive enhancement magnitude that would be sufficient to meaningfully affect kicking performance remains entirely uncertain, and effects could be too small to detect reliably or to matter practically in competitive contexts. Possible ceiling effects exist where elite players may have already optimized available proprioceptive and coordination resources through years of intensive deliberate practice, leaving minimal room for external enhancement interventions. Alternative explanations exist, where, if positive effects emerge in future research, attributing causation to specific mechanisms will require careful experimental manipulation and control conditions, and placebo effects cannot be ruled out without appropriate blinding procedures. Individual variability may occur substantially, where some players might benefit considerably, while others show no effect or even performance impairment, precluding universal application and requiring individualized assessment protocols.

Practical limitations exist as well. Optimal implementation remains unknown, where bandaging parameters, including tension magnitude, coverage area, material properties, and unilateral versus bilateral application, all require systematic empirical optimization through dedicated research. Potential adaptation effects are a concern: continuous use may lead to proprioceptive recalibration and reduced effectiveness through neural habituation, while conversely, dependency may develop, impairing performance upon sudden removal of the bandage. Regulatory uncertainty exists where competition rules regarding performance-enhancing equipment require clarification from governing bodies, and medical exception rules might not apply to performance enhancement applications, thus creating ethical and practical complications. Goalkeeper considerations exist where glove interference potential for goalkeepers might preclude application in this position despite theoretical benefits, requiring modified approaches or specialized designs for this population.

#### Integration with Existing Performance Enhancement Strategies

Hand–wrist bandaging should not be viewed in isolation but as potentially complementary to established performance enhancement strategies. Core training enhances proximal stability providing foundation for force generation while bandaging potentially enhances distal stability, optimizing terminal control, producing synergistic effects through comprehensive kinetic chain optimization. Upper-body strength training provides neuromuscular capacity for force generation, while bandaging optimizes proprioceptive control of that capacity through enhanced sensory feedback. Technical skill training develops fundamental movement patterns through repetition, while bandaging might accelerate pattern learning through enhanced proprioceptive feedback during practice.

Fatigue management demonstrates that proprioceptive acuity declines progressively with physical exhaustion [30,44,55], and bandaging might attenuate this decline through augmented mechanoreceptor stimulation, thus maintaining sensory clarity. Mental skills training produces psychological confidence effects that might interact synergistically with biomechanical mechanisms, as enhanced perceived control may improve actual motor control through psychomotor pathways [16,49]. The integration of multiple optimization strategies targeting different performance determinants may produce cumulative benefits exceeding any single intervention alone.

### 4.14. From Theory to Research: Translational Priorities

The theoretical framework presented in this review identifies hand–wrist bandaging as a candidate intervention warranting controlled scientific investigation, not as a practice-ready tool. The appropriate translational pathway moves from the present theoretical mapping, to proof-of-concept laboratory studies, to mechanistic isolation studies, to dose–response optimization, to longitudinal training studies, and only then, if a consistent efficacy signal emerges, to guided practitioner implementation with systematic monitoring.

For researchers, this review provides a detailed characterization of the theoretical rationale and a proposed research agenda. For practitioners, this review establishes only that the hypothesis is scientifically plausible and that controlled research is urgently needed. For athletes, this review provides no justification for self-experimentation, given that the effects of hand–wrist bandaging on coordination, performance, or injury risk during competitive soccer are entirely unknown.

### 4.15. Broader Implications for Sports Biomechanics

This hypothesis challenges traditional segmental approaches to performance analysis and enhancement, advocating instead for recognition that athletic movements involve integrated whole-body systems where interventions anywhere may propagate effects everywhere through complex neuromechanical coupling and proprioceptive networks spanning the entire body. While strength, power, and cardiovascular capacity receive primary attention in traditional performance enhancement paradigms, proprioceptive optimization represents an underexplored frontier potentially yielding substantial gains with minimal costs and risks, deserving greater research attention.

The majority of kinetic chain research has examined proximal-to-distal force flow, and systematic investigation of distal-to-proximal influences represents a significant and underexplored research gap in sports biomechanics. The present hypothesis contributes to this emerging line of inquiry.

## 5. Conclusions

This scoping review maps available indirect evidence from related domains that collectively support the theoretical plausibility of the hypothesis that hand–wrist bandaging may influence elite soccer kicking performance. No direct evidence exists, and all conclusions are necessarily speculative pending empirical testing.

First, upper limbs make substantial and functionally relevant contributions to kicking, including tension arc formation (which correlates strongly with ball velocity), energy transfer dominated by proximal segments, balance maintenance during the critical single-leg support phase, and kinetic chain coordination initiating force generation sequences.

Second, available evidence is consistent with bidirectional kinetic chain operation, including associations between grip function and postural control (though causality has not been established), substantial core–upper extremity correlations, and trunk stabilization that considerably enhances distal control.

Third, external joint support has been shown to enhance proprioception and neuromuscular coordination in lower and upper extremity joints, with moderate-to-large effect sizes; however, these effects have not been specifically tested for the hand–wrist complex in athletic performance contexts. Immediate effects upon application have been confirmed consistently in the available literature. Critically, no study included in this review was designed to test cross-body sensorimotor effects in the direction relevant to the present hypothesis; the plausibility of such effects rests on neurophysiological theory [18,88] and mechanoreceptor science [55,58] rather than experimental demonstration.

Despite strong theoretical foundations and robust supporting evidence from related domains, direct empirical testing of hand–wrist stabilization effects on kicking performance is completely absent from the scientific literature. This represents both the primary limitation of current knowledge and the highest-priority research opportunity deserving immediate investigation.

Hand–wrist bandaging may theoretically influence kicking performance through three proposed mechanisms: (1) enhanced sensorimotor afference improving upper limb coordination precision; (2) kinetic chain optimization through improved distal stability and force transmission efficiency; and (3) whole-body neuromechanical coordination via cross-body sensorimotor effects. Psychological confidence is noted as a secondary speculative consideration rather than a primary mechanistic pathway.

Empirical validation through rigorous controlled experimentation is necessary before any conclusions can be drawn. The theoretical foundation, while entirely built on indirect evidence, is sufficiently coherent and internally consistent to justify proof-of-concept research. If validated, this approach would contribute to a systems-based understanding of athletic performance that extends beyond traditional segment-specific models.

## Figures and Tables

**Table 1:** Characteristics and evidence classification of 51 included studies.

	Author(s), Year	Study Design	Population	Sport/Task	Primary Outcome Measure(s)	Thematic Domain	Relevance Classification
1	Lees A, Asai T, Andersen TB, Nunome H, Sterzing T. (2010) [5]	Narrative systematic review	Soccer players, elite and amateur; both sexes; mixed N; mixed age	Soccer—instep kick, side-foot kick, approach	Upper body influences on kicking technique; ball velocity determinants; arm role in balance and energy transfer	Theme 1	Direct—soccer kicking review with explicit discussion of upper body and arm contributions
2	Kellis E, Katis A. (2007) [3]	Narrative review	Soccer players; male predominant; mixed N	Soccer—instep kick	Proximal-to-distal segmental sequencing; determinants of ball speed and accuracy; upper body role	Theme 1	Direct—discusses upper limb as part of kicking biomechanical model
3	Barfield WR. (1998) [13]	Narrative review	Soccer players—developmental to elite; male; mixed N	Soccer—multiple kick types	Developmental stages of kicking; approach angle; support foot forces; upper body mechanics	Theme 1	Direct—upper body mechanics included as kicking determinant across skill levels
4	Lees A, Nolan L. (1998) [23]	Narrative review	Soccer players; male; mixed N	Soccer and related skills (kicking, throwing-in, goalkeeping)	Biomechanical factors relevant to performance; upper extremity role in kicking and balance	Theme 1	Direct—upper extremity function during kicking explicitly addressed
5	Chen J, Peek K, Sanders RH, Lee J, Pang JCY, Ekanayake K, Fu ACL. (2024) [24]	Systematic review (27 studies; n = 457)	Soccer/rugby players; mixed sex; n = 457 across studies; mixed age	Soccer—instep (n = 21), inside-of-foot (n = 1); rugby—place kick (n = 4); volley kick (n = 1)	Upper body rotation and ball-kicking performance metrics; trunk/arm contribution to ball release velocity	Theme 1	Direct—primary focus is role of upper body motions in ball-kicking performance
6	Nunome H, Asai T, Ikegami Y, Sakurai S. (2002) [2]	Cross-sectional observational (3D kinetics)	Skilled soccer players; male; small N	Soccer—side-foot and instep kicks	3D resultant joint forces and torques (lower limb); proximal-to-distal moment transfer	Theme 1	Direct (indirect for upper limb specifically)—foundational kinetic analysis; upper limb not primary outcome but provides biomechanical context
7	Fullenkamp AM, Campbell BM, Laurent CM, Lane AP. (2015) [25]	Cross-sectional observational	Collegiate soccer players; male; small N	Soccer—maximal instep kick	Trunk axial rotation and kinematics; contribution to post-strike ball velocity	Theme 1	Direct—trunk (proximal to upper limb in the kinetic chain) as a kicking determinant
8	Carvalho DDS, Ocarino JM, Cruz AC, Barsante LD et al. (2021) [26]	Cross-sectional observational (power flow analysis)	Skilled soccer players; male; small N	Soccer—maximal instep kick	Power flow from trunk to lower limb during kick; energy transfer pathways	Theme 1	Direct—power flow through trunk as energy source for kicking; directly relevant to whole-body chain
9	de Assis MA, Santos TRT, Fonseca ST, de Andrade AGP et al. (2023) [27]	Randomized Controlled Trial (n = 26)	Male participants; n = 26; adult	Soccer—instep kick	Hip kinematics (backswing phase) following resistance training of arm and anterior trunk muscles	Theme 1	Direct—RCT demonstrating non-local effect of upper limb/trunk strengthening on hip kinematics during kicking
10	Barfield WR, Kirkendall DT, Yu B. (2002) [60]	Cross-sectional observational (3D kinematics)	Elite male and female soccer players; mixed sex; n = 8	Soccer—instep kick (dominant and non-dominant)	3D kinematic differences by sex; ball velocity; upper body kinematics during kick	Theme 1	Direct—kinematic comparison including upper body parameters across sex and limb dominance
11	Zago M, Motta AF, Mapelli A, Annoni I, Galvani C, Sforza C. (2014) [28]	Cross-sectional observational (3D motion analysis)	Amateur soccer players; male; n = 9; mean age 23 yr	Soccer—inside-of-foot pass kick (preferred and non-preferred leg)	Upper limb contribution to kick biomechanics; Center of Mass kinematics; laterality differences	Theme 1	Direct—explicit 3D measurement of upper limb contribution to kicking mechanics
12	Katis A, Giannadakis E, Kannas T, Amiridis I, Kellis E, Lees A. (2013) [29]	Cross-sectional observational (EMG + kinematics)	Soccer players; male; adult	Soccer—instep kick (accuracy conditions)	EMG activity and segmental kinematics influencing kick accuracy; muscle coordination	Theme 1	Direct—neuromuscular coordination during kicking; upper body’s role in accuracy
13	Apriantono T, Nunome H, Ikegami Y, Sano S. (2006) [30]	Cross-sectional experimental (fatigue protocol)	Soccer players; male; small N; adult	Soccer—instep kick (before and after fatigue)	Instep kicking kinetics and kinematics under muscle fatigue; inter-segmental coordination degradation	Theme 1	Direct—fatigue-induced disruption of whole-body kick coordination, including upper–lower limb inter-segmental effects
14	Chu SK, Jayabalan P, Kibler WB, Press J. (2016) [12]	Narrative review	Overhead throwing athletes; male predominant	Baseball pitching; tennis serve	Kinetic chain mechanics; proximal-to-distal energy sequencing; clinical evaluation of kinetic chain deficits	Theme 2	Indirect-Biomechanical—kinetic chain principles from overhead sports; translational framework for bidirectional limb linkage
15	Fleisig GS, Escamilla RF, Andrews JR et al. (1996) [31]	Cross-sectional observational	Elite baseball pitchers and football quarterbacks; male; adult	Baseball pitch vs. football pass	Kinematic and kinetic parameters; wrist flexion in kinetic chain sequence; segmental sequencing	Theme 2	Indirect-Biomechanical—wrist as terminal segment in kinetic chain; proximal-to-distal energy transfer
16	Seroyer ST, Nho SJ, Bach BR et al. (2010) [32]	Narrative review	Overhead throwing athletes; baseball pitchers; male	Baseball pitching	Kinetic chain role in performance enhancement and injury prevention; proximal segment contributions to distal output	Theme 2	Indirect-Biomechanical—kinetic chain model in throwing: proximal stability enabling distal performance
17	Hirashima M, Kadota H, Sakurai S, Kudo K, Ohtsuki T. (2003) [61]	Observational (EMG + 3D motion capture)	Trained throwers; male; small N	Overarm throw	Sequential muscle activation in upper extremity and trunk; timing and coordination of proximal-to-distal muscle recruitment	Theme 2	Indirect-Biomechanical—demonstrates sequential muscle activation cascade that includes distal upper limb segments
18	Fortenbaugh D, Fleisig GS, Andrews JR. (2009) [33]	Narrative review	Baseball pitchers; male	Baseball pitching	Biomechanical performance indicators; kinematic and kinetic risk factors; wrist and hand mechanics in follow-through	Theme 2	Indirect-Biomechanical—distal segment (wrist/hand) as part of pitching biomechanics review
19	Fleisig G, Nicholls R, Elliott B, Escamilla R. (2003) [34]	Cross-sectional observational	Tennis players; mixed sex; adult	Tennis serve	Joint loads in upper extremity during different serve techniques; trunk–arm loading relationship	Theme 2	Indirect-Biomechanical—upper limb loading in striking sport; kinetic chain from trunk to distal arm
20	Weber AE, Kontaxis A, O’Brien SJ, Bedi A. (2014) [35]	Narrative review	Throwing athletes; mixed	Overarm throw (general)	Simplified biomechanical overview of throwing; kinetic chain function; wrist as terminal force-output segment	Theme 2	Indirect-Biomechanical—accessible overview of throwing chain, including wrist’s role
21	Wilke J, Krause F. (2019) [36]	Systematic review of anatomical studies	Cadaveric/anatomical studies; n/a	n/a—anatomical connectivity	Myofascial chain anatomy of the upper limb; cross-body fascial connections	Theme 2	Indirect-Biomechanical—anatomical basis for myofascial connectivity linking upper limb to trunk and lower limb
22	Kibler WB. (2000) [37]	Narrative review/Expert opinion	Sports injury rehabilitation patients; mixed	Multiple sports (throwing, overhead)	Closed kinetic chain principles in rehabilitation; proximal-to-distal and distal-to-proximal force transfer	Theme 2	Indirect-Biomechanical—closed kinetic chain model: distal stabilization affects proximal performance
23	Solomito MJ, Garibay EJ, Woods JR et al. (2015) [38]	Cross-sectional observational	Baseball pitchers; male; adolescent and adult	Baseball pitching	Effect of lateral trunk lean on ball velocity and upper extremity joint moments; trunk-upper limb interaction	Theme 2	Indirect-Biomechanical—trunk–upper limb mechanical coupling affecting both performance and joint loading
24	Ghai S, Ghai I, Narciss S. (2024) [16]	Systematic review + meta-analysis (91 studies; n = 2718)	Mixed populations (healthy, ankle instability, stroke, OA); both sexes; n = 2718	Multiple joints (ankle, knee, shoulder, wrist); multiple tasks	Repositioning error (JPS); threshold to detect passive motion; active movement extent; taping vs. no tape and vs. placebo	Theme 3	Indirect-Proprioceptive—largest meta-analysis to date on taping and proprioception; provides broad mechanistic support
25	Ghai S, Ghai I, Narciss S. (2023) [65]	Systematic review + meta-analysis (11 studies; n = 279)	Mixed populations (healthy and injured); both sexes; n = 279	Multiple joints; force-matching tasks	Absolute and relative force sense accuracy; taping vs. no comparator and vs. placebo tape	Theme 3	Indirect-Proprioceptive—meta-analytic evidence for taping and kinetic proprioception (force sense)
26	Raymond J, Nicholson LL, Hiller CE, Refshauge KM (2012) [66]	Systematic review + meta-analysis (8 studies)	Athletes/patients with functional ankle instability; mixed sex	Ankle—proprioception testing (JPS and kinesthesia)	Proprioceptive acuity (JPS, movement detection) with and without ankle tape/brace	Theme 3	Indirect-Proprioceptive—pooled evidence on ankle taping and proprioception; finding: no significant effect in FAI population (conflicting with healthy populations)
27	Heß T, Milani TL, Kilper A, Mitschke C. (2024) [62]	RCT	Subacute ankle sprain patients; mixed sex; small N; adult	Ankle—balance, gait, fine ankle coordination tasks (foot pedal)	Single-leg balance; gait parameters; ankle fine motor coordination (foot-pedal task)	Theme 3	Indirect-Proprioceptive.
28	Ucuzoglu ME, Unver B, Sarac DC, Cilga G. (2020) [39]	RCT (n = 68)	Healthy adults; mixed sex; n = 68	Wrist—angle reproduction task (JPS)	Wrist joint position sense with taping vs. elastic bandaging; improvements at 20 min and 24 h post-application	Theme 3	Indirect-Proprioceptive—direct evidence that wrist external support improves JPS in healthy individuals
29	Justo-Cousiño LA, Da Cuña-Carrera I, Alonso-Calvete A, González-González Y. (2024) [40]	RCT	Healthy subjects; mixed sex; adult	Wrist—JPS and force sense testing	Kinesio Tape effects on wrist JPS and force sense; significant improvement only in JPS at 30° extension	Theme 3	Indirect-Proprioceptive—RCT on KT and wrist proprioception; note: partial effect only (extension JPS)
30	Özen Oruk D, Karakaya MG, Yenişehir S, Çıtak Karakaya İ. (2023) [41]	RCT	Patients with wrist pathology; mixed sex; adult	Wrist—kinematics (goniometry) and functional performance	KT on wrist flexor (FCU) or extensor (ECRB/L) muscles; wrist ROM and functional performance	Theme 3	Indirect-Proprioceptive—RCT demonstrating KT application to wrist muscles improves kinematics and performance
31	Lin ZM, Yang JF, Lin YL, Cheng YC, Hung CT, Chen CS, Chou LW. (2021) [42]	Crossover experimental study (n = 24)	Healthy adults; mixed sex; n = 24; 20–40 yr	Hand/wrist—force control, JPS, reaction time, motor cortex activity (EEG)	KT tension effects on hand sensorimotor control; JPS angle error; reaction time; cortical activity (EEG)	Theme 3	Indirect-Proprioceptive—neurophysiological evidence for cutaneous feedback via taping modulating central sensorimotor processing
32	Miralles I, Monterde S, Montull S, Salvat I, Fernández-Ballart J, Beceiro J. (2010) [43]	RCT (crossover)	Healthy volunteers; mixed sex; n = 40; mean age 23 yr	Ankle—3D joint position sense testing	JPS (absolute error between estimated and target angles); ankle taping for lateral ligament sprain	Theme 3	Indirect-Proprioceptive—taping improves proprioception in healthy (non-injured) volunteers; relevant to healthy athlete context
33	Jahjah A, Seidenspinner D, Schüttler K et al. (2018) [44]	RCT	Healthy subjects; mixed sex; adult	Ankle—JPS under fatigue conditions	Ankle JPS after local muscle fatigue with and without tape; tape preserves JPS under fatigue	Theme 3	Indirect-Proprioceptive—taping maintains proprioception under fatigue; relevant to end-of-game performance context
34	Alawna M, Mohamed AA. (2020) [45]	RCT	Volleyball players with chronic ankle instability; mixed sex; adult	Ankle—balance, proprioception, vertical jump	Short-term and long-term effects of taping and bandaging on balance, JPS, and vertical jump performance	Theme 3	Indirect-Proprioceptive—bandaging (not only tape) shown to affect proprioception and functional performance in athletes
35	Yen SC, Folmar E, Friend KA, Wang YC. (2018) [67]	Cross-sectional observational	Chronic ankle instability patients; mixed sex; adult	Ankle—walking (gait analysis)	Ankle kinematics (angles) during walking with athletic tape vs. kinesiology tape vs. no tape	Theme 3	Indirect-Proprioceptive—external ankle support modifies gait kinematics
36	Kacmaz KS, Unver B. (2024) [46]	Single-blind RCT (placebo-controlled)	Healthy individuals; mixed sex; adult	Elbow—JPS testing	KT effects on elbow joint position sense vs. placebo tape	Theme 3	Indirect-Proprioceptive—extends taping–proprioception evidence to upper limb joint (elbow); mechanistically adjacent to wrist
37	Bravi R, Quarta E, Cohen EJ, Gottard A, Minciacchi D. (2014) [47]	Crossover experimental study	Healthy adults; mixed sex; small N	Upper limb—rhythmic motor tasks (finger/wrist movements)	Kinesiotaping of the motor effector; neural mechanisms for rhythmic movements; movement variability	Theme 3	Indirect-Proprioceptive—mechanistic evidence for KT on upper limb neural control; distal taping modulates central motor patterns
38	Annino G, Alashram AR, Romagnoli C et al. (2023) [48]	RCT crossover	Healthy soccer players; male; adult	Soccer—functional performance tests (CMJ, sprint, and agility)	Acute effects of KT (lower limb application) on functional performance in soccer	Theme 3	Indirect-Proprioceptive—closest study to target population; tests KT in soccer players but addresses lower limb taping, not hand–wrist; no kicking-specific outcomes
39	Dehghan F, Fouladi R, Martin J. (2024) [49]	Scoping review (50 studies)	Athletes and physically active individuals; mixed sex	Multiple sports; multiple joints	KT effects on pain, performance/function, strength, proprioception/balance, injury prevention in athletes	Theme 3	Indirect-Proprioceptive—broad overview of KT evidence in sport: 54% of studies found no effect; 46% found some supporting evidence
40	Cho HY, Kim EH, Kim J, Yoon YW. (2015) [50]	RCT	Older patients with knee osteoarthritis; mixed sex; ≥55 yr	Knee—pain, ROM, proprioception (JPS)	KT effects on pain (VAS), knee ROM (goniometry), and proprioception (JPS angle error); KT superior to control	Theme 3	Indirect-Proprioceptive—RCT evidence for KT improving proprioception at a major joint; non-athletic, clinical population limits transferability
41	Werle S, Goldhahn J, Drerup S et al. (2009) [51]	Cross-sectional normative data study	Healthy Swiss adults; both sexes; large N; 20–94 yr	Hand—grip and pinch dynamometry (standardized test position)	Age- and sex-stratified normative reference values for grip and pinch strength	Theme 4	Indirect-Distal NOTE: This is a normative data study only.
42	Bohannon RW. (2019) [52]	Narrative review	Older adults; mixed sex; multiple studies	Hand—grip dynamometry (multiple contexts)	HGS as biomarker of overall health and functional capacity; associations with muscle mass, mortality, and disability	Theme 4	Indirect-Distal—contextualizes HGS as a systemic strength indicator, not sport-specific
43	Bohannon RW. (2012) [53]	Cross-sectional observational	Adults; mixed sex; mixed N	Hand—grip dynamometry; knee—isokinetic dynamometry	Correlation between grip strength and knee extension strength; common construct hypothesis	Theme 4	Indirect-Distal—assesses whether grip strength reflects global muscle strength; relevant to hand–wrist stability as a proxy for whole-body function
44	Takemura RL, Ortolani CC, Saito M et al. (2023) [54]	Observational crossover study	CrossFit athletes; mixed sex; adult	CrossFit—handgrip dynamometry test	Effect of wrist wrap on handgrip strength in CrossFit athletes	Theme 4	Indirect-Distal—most direct evidence that a wrist external support (wrap) can modify grip strength in athletes; non-kicking sport
45	Hagert E, Rein S. (2024) [55]	Narrative review	N/A (review of mechanoreceptor and clinical literature)	Wrist—clinical assessment and rehabilitation tasks	Wrist mechanoreceptor anatomy (Ruffini, Pacini, and Meissner); proprioceptive pathways; clinical implications of wrist proprioception for rehabilitation	Theme 4	Indirect-Distal—foundational neurophysiological basis for wrist proprioceptive mechanisms; relevant to theoretical rationale
46	Sánchez-Montoya LJ, Sánchez DP, Ordoñez-Mora LT. (2023) [56]	Scoping review	Posttraumatic wrist injury patients; mixed sex	Wrist—proprioceptive rehabilitation tasks	Proprioceptive rehabilitation strategies in wrist injuries; evidence base for proprioceptive training	Theme 4	Indirect-Distal—proprioceptive wrist rehabilitation literature; establishes clinical importance of wrist proprioception
47	Hong SJ, Lee MY, Lee BH. (2024) [64]	RCT (n = 31)	Patients with nonspecific chronic wrist pain; mixed sex; n = 31; adult	Wrist—pain, ROM, grip strength, functional performance	Wrist stability training combined with grip strengthening: effects on pain, function, and ROM	Theme 4	Indirect-Distal—wrist stability training improves grip and function; informs wrist stabilization principles
48	O’Driscoll SW, Horii E, Ness R et al. (1992) [57]	Experimental study	Healthy subjects; mixed sex; n = 20; adult	Wrist—grip dynamometry at varying wrist positions	Relationship between wrist position (flexion/extension/deviation), grasp size and grip strength	Theme 4	Indirect-Distal—wrist position directly affects grip force output; foundational biomechanics of wrist–hand function
49	Hagert E. (2010) [58]	Narrative review	N/A (review)	Wrist—clinical and experimental proprioception literature	Mechanoreceptors in wrist ligaments; neural pathways; wrist proprioception impairment and rehabilitation implications	Theme 4	Indirect-Distal—earlier foundational review by same senior author (Hagert) establishing wrist proprioception science
50	Wu CK, Lin YC, Lai CP, Wang HP, Hsieh TH. (2022) [59]	RCT	Young volleyball athletes; mixed sex; adult	Volleyball—drop landing task (biomechanical analysis)	Landing biomechanics (ground reaction forces, joint angles) with dynamic taping vs. no taping	Theme 4	Indirect-Distal—dynamic taping applied to lower extremity improves landing mechanics in athletes; broader taping-performance evidence
51	Nesser TW, Huxel KC, Tincher JL, Okada T. (2008) [63]	Cross-sectional observational	Division I American football players; male; n = 23; mean age 20 yr	American football—core stability tests; sprint; vertical jump; 3-cone agility	Relationship between core stability (various tests) and athletic performance (speed, power, and agility)	Theme 4	Indirect-Distal—core-to-distal performance relationship

## Data Availability

All data supporting the findings of this scoping review are available within the article. The complete search strategies, inclusion and exclusion criteria, and data extraction templates are available from the corresponding author upon reasonable request.

## Supplementary Materials

The following supporting information can be downloaded at: https://www.mdpi.com/article/10.3390/sports14050189/s1, PRISMA 2020 checklist.

## Abbreviations

The following abbreviations are used in this manuscript:

PRISMA-ScR	Preferred Reporting Items for Systematic Reviews and Meta-Analyses Extension for Scoping Reviews
RCT	Randomized Controlled Trial
EMG	Electromyography
MeSH	Medical Subject Headings
3D	Three-dimensional
sEMG	Surface electromyography
