# Supplementary material for: Why Hand–Wrist Bandaging Could Improve Performance in Elite Soccer Players? A Scoping Review on the Biomechanical Rationale of Upper Limb Role in Kicking
Source: Sports (Basel). 2026 May 6;14(5):189. doi: 10.3390/sports14050189 (PMC13211177; doi:10.3390/sports14050189)
Supplement: Supplementary file 1 [file sports-14-00189-s001.zip › PRISMA-2009-Flow-Diagram.pdf]

PRISMA 2009 Flow Diagram

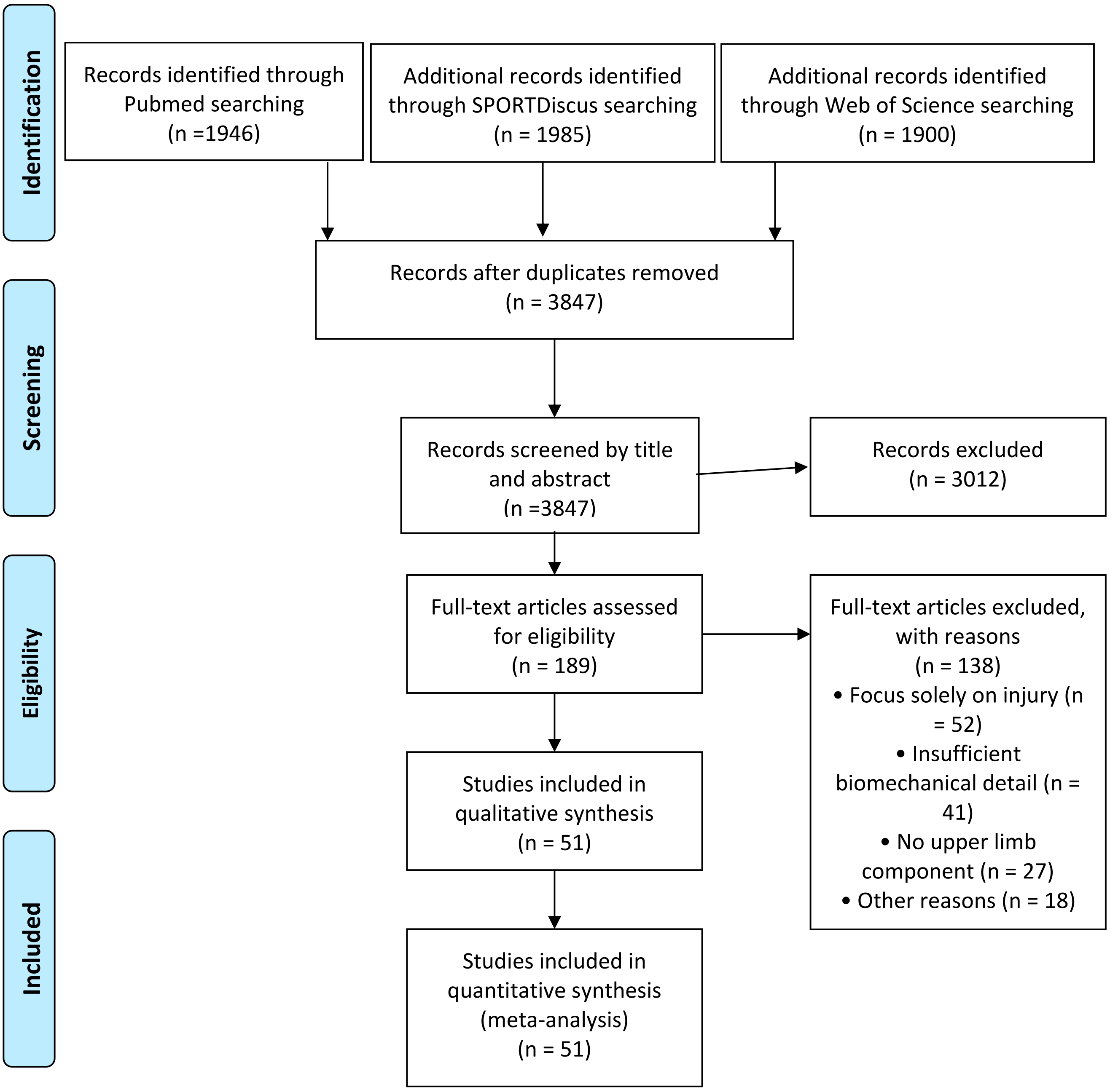

From: Moher D, Liberati A, Tetzlaff J, Altman DG, The PRISMA Group (2009). *P*referred *R*eporting *I*tems for *S*ystematic Reviews and *M*eta-*A*nalyses: The PRISMA Statement. PLoS Med 6(6): e1000097. doi:10.1371/journal.pmed1000097

For more information, visit www.prisma-statement.org.
