# Supplementary material for: Why Hand–Wrist Bandaging Could Improve Performance in Elite Soccer Players? A Scoping Review on the Biomechanical Rationale of Upper Limb Role in Kicking
Source: Sports (Basel). 2026 May 6;14(5):189. doi: 10.3390/sports14050189 (PMC13211177; doi:10.3390/sports14050189)
Supplement: Supplementary file 1 [file sports-14-00189-s001.zip › PRISMA_2020_checklist.pdf]

PRISMA 2020 Checklist

Section and Topic	Item #	Checklist item	Location where item is reported
**TITLE**			
Title	1	Identify the report as a systematic review.	Title clearly identifies this as a "scoping review"
**ABSTRACT**			
Abstract	2	See the PRISMA 2020 for Abstracts checklist.	Abstract section (page 1) includes: Background, Methods (databases, guidelines), Results (number of studies, date range, key findings), and Conclusions
**INTRODUCTION**			
Rationale	3	Describe the rationale for the review in the context of existing knowledge.	ntroduction, paragraphs 1-5 (pages 1-3). Describes evolution from 2D to 3D analysis, kinetic chain concepts, upper limb roles, and research gap
Objectives	4	Provide an explicit statement of the objective(s) or question(s) the review addresses.	Introduction, final paragraph before Section 2 (page 3). Three explicit research questions provided
**METHODS**			
Eligibility criteria	5	Specify the inclusion and exclusion criteria for the review and how studies were grouped for the syntheses.	Section 2.3 Eligibility Criteria (page 4). Inclusion criteria for study types, populations, and topics specified. Exclusion criteria detailed. Section 2.5 describes thematic grouping into four categories
Information sources	6	Specify all databases, registers, websites, organisations, reference lists and other sources searched or consulted to identify studies. Specify the date when each source was last searched or consulted.	Section 2.2 Information Sources and Search Strategy (pages 3-4). Databases: PubMed/MEDLINE (1946 onwards), Web of Science Core Collection (1900 onwards), SPORTDiscus (1985 onwards), Google

PRISMA 2020 Checklist

Section and Topic	Item #	Checklist item	Location where item is reported
			Scholar. Search date: through February 21, 2026
Search strategy	7	Present the full search strategies for all databases, registers and websites, including any filters and limits used.	Section 2.2 Information Sources and Search Strategy (pages 4-5). Five conceptual domains detailed with specific search terms and Boolean operators. Combined comprehensive search string provided
Selection process	8	Specify the methods used to decide whether a study met the inclusion criteria of the review, including how many reviewers screened each record and each report retrieved, whether they worked independently, and if applicable, details of automation tools used in the process.	Section 2.4 Selection Process (page 5). Two reviewers independently screened titles/abstracts and full-text articles. Disagreements resolved through discussion or third reviewer consultation. Reference list hand-searching and forward citation tracking performed
Data collection process	9	Specify the methods used to collect data from reports, including how many reviewers collected data from each report, whether they worked independently, any processes for obtaining or confirming data from study investigators, and if applicable, details of automation tools used in the process.	Section 2.5 Data Extraction and Analysis (pages 5-6). Standardized template used. One reviewer extracted data, second reviewer verified 20% random sample (94% agreement). No automation tools mentioned
Data items	10a	List and define all outcomes for which data were sought. Specify whether all results that were compatible with each outcome domain in each study were sought (e.g. for all measures, time points, analyses), and if not, the methods used to decide which results to collect.	Section 2.5 Data Extraction and Analysis (page 5). Data items include: study characteristics, methodological details, key findings (quantitative results, effect sizes, statistical significance), thematic classifications, and quality indicators

PRISMA 2020 Checklist

Section and Topic	Item #	Checklist item	Location where item is reported
	10b	List and define all other variables for which data were sought (e.g. participant and intervention characteristics, funding sources). Describe any assumptions made about any missing or unclear information.	Section 2.5 Data Extraction and Analysis (page 5). Variables include: sample size, population characteristics (skill level, age, gender), motion capture specifications, EMG protocols, force platforms. No specific assumptions about missing data mentioned
Study risk of bias assessment	11	Specify the methods used to assess risk of bias in the included studies, including details of the tool(s) used, how many reviewers assessed each study and whether they worked independently, and if applicable, details of automation tools used in the process.	Section 2.6 Quality Assessment (page 6). Explicitly states no formal quality assessment performed, consistent with scoping review methodology. Methodological rigor noted during data extraction including 3D vs 2D capture, sample size, control groups
Effect measures	12	Specify for each outcome the effect measure(s) (e.g. risk ratio, mean difference) used in the synthesis or presentation of results.	Not applicable - scoping review with narrative synthesis. Various effect measures reported from included studies (correlations, percentages, effect sizes) but no standardized effect measure applied across studies
Synthesis methods	13a	Describe the processes used to decide which studies were eligible for each synthesis (e.g. tabulating the study intervention characteristics and comparing against the planned groups for each synthesis (item #5)).	Section 2.5 Data Extraction and Analysis (page 6). Studies grouped into four primary themes based on conceptual domains. Narrative synthesis approach employed given heterogeneous study designs
	13b	Describe any methods required to prepare the data for presentation or synthesis, such as handling of missing summary statistics,	Not explicitly addressed. Scoping review

Section and Topic	Item #	Checklist item	Location where item is reported
		or data conversions.	methodology does not require data conversions or statistical preparation
	13c	Describe any methods used to tabulate or visually display results of individual studies and syntheses.	Section 3.1 mentions PRISMA-ScR flow diagram (Figure 1). Narrative synthesis organized by themes described in Section 2.5
	13d	Describe any methods used to synthesize results and provide a rationale for the choice(s). If meta-analysis was performed, describe the model(s), method(s) to identify the presence and extent of statistical heterogeneity, and software package(s) used.	Section 2.5 Data Extraction and Analysis (page 6). Narrative synthesis approach explicitly chosen due to heterogeneous study designs and exploratory scoping review purpose. No meta-analysis performed
	13e	Describe any methods used to explore possible causes of heterogeneity among study results (e.g. subgroup analysis, meta-regression).	Not applicable - narrative synthesis only, no quantitative heterogeneity analysis
	13f	Describe any sensitivity analyses conducted to assess robustness of the synthesized results.	Not applicable for scoping review with narrative synthesis
Reporting bias assessment	14	Describe any methods used to assess risk of bias due to missing results in a synthesis (arising from reporting biases).	Section 4.9 Limitations (page 27) acknowledges publication bias as a limitation but states no formal assessment performed. Notes that systematic reviews showed high proportions of positive effects
Certainty assessment	15	Describe any methods used to assess certainty (or confidence) in the body of evidence for an outcome.	Not performed. Acknowledged in Section 4.9 as limitation of scoping review methodology
**RESULTS**			
Study selection	16a	Describe the results of the search and selection process, from the number of records identified in the search to the number of studies included in the review, ideally using a flow diagram.	Section 3.1 Search Results and Study Selection (page 6).

Section and Topic	Item #	Checklist item	Location where item is reported
			Details provided: 3,847 unique records identified, 3,012 excluded after title/abstract screening, 189 full-text assessed, 138 excluded with reasons, 26 added from references, 51 final included. PRISMA-ScR flow diagram (Figure 1) provided
	16b	Cite studies that might appear to meet the inclusion criteria, but which were excluded, and explain why they were excluded.	Section 3.1 (page 6). Exclusion reasons provided: injury focus without performance (n=52), insufficient biomechanical detail (n=41), no upper limb component (n=27). Individual citations not provided
Study characteristics	17	Cite each included study and present its characteristics.	Section 3.2 Study Characteristics (pages 6-7). Aggregate characteristics provided: date range (1988-2025), countries, study designs, sample sizes, skill levels, gender distribution, measurement technologies. Individual studies cited throughout Sections 3.3-3.6 with specific details
Risk of bias in studies	18	Present assessments of risk of bias for each included study.	Not performed. Section 2.6 states no formal quality assessment consistent with scoping review methodology. Methodological limitations noted qualitatively during synthesis
Results of individual studies	19	For all outcomes, present, for each study: (a) summary statistics for each group (where appropriate) and (b) an effect estimate and its precision (e.g. confidence/credible interval), ideally using structured tables or plots.	Sections 3.3-3.6 (pages 7-17). Individual study findings presented narratively with specific

Section and Topic	Item #	Checklist item	Location where item is reported
			quantitative results (correlations, percentages, effect sizes, p-values) when reported in original studies. No structured tables of individual study results provided
Results of syntheses	20a	For each synthesis, briefly summarise the characteristics and risk of bias among contributing studies.	Sections 3.3-3.6 provide narrative summaries by theme. Study characteristics summarized in Section 3.2. Risk of bias not formally assessed but methodological quality noted
	20b	Present results of all statistical syntheses conducted. If meta-analysis was done, present for each the summary estimate and its precision (e.g. confidence/credible interval) and measures of statistical heterogeneity. If comparing groups, describe the direction of the effect.	Not applicable - no statistical syntheses or meta-analysis performed. Narrative synthesis only
	20c	Present results of all investigations of possible causes of heterogeneity among study results.	Not applicable - narrative synthesis without formal heterogeneity analysis
	20d	Present results of all sensitivity analyses conducted to assess the robustness of the synthesized results.	Not applicable - no sensitivity analyses performed
Reporting biases	21	Present assessments of risk of bias due to missing results (arising from reporting biases) for each synthesis assessed.	Not performed. Acknowledged as limitation in Section 4.9 (page 27)
Certainty of evidence	22	Present assessments of certainty (or confidence) in the body of evidence for each outcome assessed.	Not performed. Acknowledged as limitation of scoping review methodology in Section 4.9
**DISCUSSION**			
Discussion	23a	Provide a general interpretation of the results in the context of other evidence.	Section 4.1 Summary of Key Evidence (page 17). Section 4.2 Critical Research Gap (pages 17-18). Section 3.7 Integrated Synthesis (pages 15-17) interprets

PRISMA

PRISMA 2020 Checklist

Section and Topic	Item #	Checklist item	Location where item is reported
			findings across themes
	23b	Discuss any limitations of the evidence included in the review.	Section 4.9 Limitations (pages 26-28). Comprehensive discussion of evidence limitations including no direct studies, cross-joint extrapolation, sport generalization, elite population underrepresentation
	23c	Discuss any limitations of the review processes used.	Section 4.9 Limitations (pages 26-27). Methodological limitations including no formal quality assessment, no meta-analysis, potential interpretation bias, publication bias
	23d	Discuss implications of the results for practice, policy, and future research.	Section 4.10 Future Research Directions (pages 21-24), Section 4.11 Methodological Recommendations (pages 24-25), Section 4.12 Implications for Sports Medicine (pages 25-26), Section 4.14 Knowledge Translation (pages 28-29)
**OTHER INFORMATION**			
Registration and protocol	24a	Provide registration information for the review, including register name and registration number, or state that the review was not registered.	Section 2.1 Protocol and Registration (page 3). States "The review protocol was not pre-registered, consistent with exploratory scoping review methodology"
	24b	Indicate where the review protocol can be accessed, or state that a protocol was not prepared.	Section 2.1 (page 3). States protocol was not prepared, consistent with scoping review methodology
	24c	Describe and explain any amendments to information provided at registration or in the protocol.	Not applicable - no registration or protocol

PRISMA 2020 Checklist

Section and Topic	Item #	Checklist item	Location where item is reported
Support	25	Describe sources of financial or non-financial support for the review, and the role of the funders or sponsors in the review.	Funding section (page 32). States "This research received no external funding"
Competing interests	26	Declare any competing interests of review authors.	Conflicts of Interest section (page 32). States "The authors declare no conflicts of interest"
Availability of data, code and other materials	27	Report which of the following are publicly available and where they can be found: template data collection forms; data extracted from included studies; data used for all analyses; analytic code; any other materials used in the review.	Data Availability Statement (page 32). States "All data supporting the findings of this scoping review are available within the article. The complete search strategies, inclusion and exclusion criteria, and data extraction templates are available from the corresponding author upon reasonable request"

From: Page MJ, McKenzie JE, Bossuyt PM, Boutron I, Hoffmann TC, Mulrow CD, et al. The PRISMA 2020 statement: an updated guideline for reporting systematic reviews. BMJ 2021;372:n71. doi: 10.1136/bmj.n71.
This work is licensed under CC BY 4.0. To view a copy of this license, visit https://creativecommons.org/licenses/by/4.0/
